# *FCGR2B* ^+^ Macrophages as a Critical Node Linking Ferroptosis and Immunosuppression: A Multiomics Framework for Prognosis and Therapy in High‐Grade Serous Ovarian Cancer

**DOI:** 10.1155/humu/8027584

**Published:** 2026-04-06

**Authors:** Jialu Zhou, Tao Zeng, Yi Liu, Mingxia Ye, Mingxia Li, Zhe Zhang, Yuanguang Meng

**Affiliations:** ^1^ Medical School of Chinese PLA, Beijing, China, 301hospital.com.cn; ^2^ Department of Gynecology and Obstetrics, 7th Medical Center of Chinese PLA General Hospital, Beijing, China; ^3^ Faculty of Hepato-Biliary-Pancreatic Surgery, 1st Medical Center of Chinese PLA General Hospital, Beijing, China; ^4^ Department of General Surgery, 7th Medical Center of Chinese PLA General Hospital, Beijing, China

**Keywords:** *FCGR2B*, ferroptosis, high-grade serous ovarian cancer, macrophage, tumor microenvironment

## Abstract

**Background:**

High‐grade serous ovarian cancer (HGSOC) is characterized by a complex tumor microenvironment and poor prognosis, yet the roles of specific tumor‐associated macrophages (TAMs) subpopulations in driving disease progression remain elusive.

**Methods:**

This study evaluated the prognostic relevance of *FCGR2B* in HGSOC. Single‐cell RNA sequencing identified *FCGR2B* ^+^ TAMs as a distinct macrophage subpopulation with unique transcriptional features. Integrative analyses combining single‐cell and bulk differentially expressed genes, macrophage‐associated modules, and ferroptosis‐related gene sets identified 26 candidate prognostic genes, from which a four‐gene signature (*CRYAB*, *PLAUR*, *EREG*, and *C5AR1*) was derived to construct the prognostic risk model. The model was validated in an independent cohort. Immune infiltration, single‐cell trajectory, copy number variation, and drug–gene associations were analyzed to explore the molecular and therapeutic implications of risk stratification.

**Results:**

HGSOC patients classified as high risk exhibited poorer survival outcomes, increased infiltration of M2‐like macrophages, elevated expression of immune checkpoints, and enrichment of immune‐ and ferroptosis‐related pathways. Trajectory and copy number variation analyses revealed stage‐specific gene expression patterns and amplification‐associated regulation. Drug–gene association analyses further suggested that high‐risk patients may be more responsive to targeted therapies and proteasome inhibitors, whereas low‐risk patients may benefit from conventional chemotherapy.

**Conclusion:**

*FCGR2B* ^+^ TAMs are closely linked to HGSOC progression, and the proposed prognostic model based on *FCGR2B* ^+^ TAMs provides predictive value and potential therapeutic insights for patient stratification.

## 1. Introduction

High‐grade serous ovarian cancer (HGSOC) is the most prevalent and lethal subtype of epithelial ovarian cancer (EOC), characterized by high recurrence and chemoresistance [1–3]. The tumor microenvironment (TME) of HGSOC is highly heterogeneous, encompassing diverse immune cell states and dysfunctional antitumor immunity [4–7]. Among immune cell populations, tumor‐associated macrophages (TAMs) play a dual role, contributing to antitumor immunity but frequently being reprogrammed to promote tumor growth, angiogenesis, and chemoresistance [8, 9]. Recent studies highlight the significant metabolic plasticity of TAMs, whose glucose, amino acid, and lipid metabolism are reprogrammed within the TME to dictate their polarization and functional phenotypes [10]. Metabolic reprogramming in TAMs not only fuels their immunosuppressive functions but also orchestrates immune evasion and metabolic crosstalk, which are now essential for understanding TAM heterogeneity and their roles in cancer [11]. Understanding these metabolic networks is therefore crucial for elucidating TAM heterogeneity and their roles in tumor progression [12]. This functional plasticity makes TAMs attractive therapeutic targets; however, the molecular insights underlying the reprogramming of specific TAM subsets and their clinical relevance in HGSOC remain poorly understood.

Among immunosuppressive TAM subsets, inhibitory Fc receptor–expressing macrophages, exemplified by Fc gamma receptor IIB (Fc*γ*RIIB, encoded by *FCGR2B*), have been reported to promote immune evasion and therapeutic resistance across multiple cancers [13–15]. Mechanistically, *FCGR2B* ^+^ TAMs can activate STAT3‐dependent inflammatory signaling through extracellular vesicles and downstream cytokine cascades, thereby enhancing tumor stemness and fostering an immunosuppressive microenvironment in colorectal liver metastases [13]. However, their transcriptional programs, functional heterogeneity, and clinical relevance remain largely unexplored in HGSOC.

Ferroptosis, an iron‐dependent form of regulated cell death, has been shown to influence tumor progression and the immune microenvironment in OC, with high‐risk patients exhibiting increased M2 macrophage infiltration and immune checkpoint expression [16]. Given that ferroptosis can modulate macrophage polarization and immune interactions in OC [17–19], it is plausible that *FCGR2B* ^+^ TAMs may exhibit distinct ferroptosis‐related transcriptional programs contributing to their immunosuppressive function in HGSOC.

This study integrated single‐cell and bulk transcriptomic data to characterize *FCGR2B* ^+^ TAMs in HGSOC. These macrophages showed enrichment of ferroptosis‐related gene sets, and a four‐gene prognostic model was developed to stratify patients based on overall survival (OS). High‐risk patients were predicted to exhibit increased immunosuppressive features and may respond better to targeted therapies and proteasome inhibitors, whereas low‐risk patients may be more sensitive to conventional chemotherapy. This study provides a cell type–resolved framework for understanding TAM heterogeneity and ferroptosis‐associated immunoregulation in HGSOC, suggesting potential therapeutic targets for further investigation.

## 2. Materials and Methods

### 2.1. Data Collection

RNA‐seq data from untreated HGSOC and normal ovarian tissues were obtained from the TCGA‐OV (https://portal.gdc.cancer.gov/) and the Genotype‐Tissue Expression (GTEx) project, respectively, and used as the training set (399 HGSOC [20] and 193 normal). An independent validation cohort of untreated HGSOC patients was obtained from GSE102073 [21] (*n* = 83). Single‐cell RNA‐seq data from HGSOC (*n* = 7) and age‐matched nonmalignant ovarian tissues (*n* = 5) were obtained from GSE184880 [4]. Ferroptosis‐related genes (FRGs, *n* = 2023) were retrieved from GeneCards using the keyword “Ferroptosis”.

### 2.2. scRNA‐seq Analysis

Single‐cell RNA‐seq data were processed using Seurat v5.1.0. Genes expressed in fewer than three cells were excluded, and high‐quality cells were retained if they had 200–4000 detected genes, total counts between 500 and 15,000, and < 25% mitochondrial gene expression. The data were log‐normalized and scaled, and the Top 2000 highly variable genes were identified for downstream analysis. Principal component analysis (PCA) was performed, and the significance of principal components was evaluated using JackStraw and ElbowPlot analyses. Cells were clustered using the Top 15 PCs with the FindNeighbors and FindClusters functions (resolution = 0.5), with batch effects corrected using Harmony. Uniform Manifold Approximation and Projection (UMAP) was applied for dimensionality reduction and visualization. Cluster‐specific marker genes (min.pct = 0.5, log fold − change > 0.25, adjusted *p* < 0.05) were identified. Cell types were assigned by comparing these markers with previous literature [4] and the CellMarker database. The final annotation of cell types and their markers is summarized in Table S1.

### 2.3. High‐Dimensional Weighted Gene Coexpression Network (HdWGCNA) Analysis

Macrophage‐weighted gene coexpression networks were constructed using hdWGCNA (v0.4.00). To mitigate sparsity, metacells were generated by aggregating similar cells. A total of 14 coexpression modules were identified using dynamic tree cutting, of which 12 modules were highly expressed in macrophages and defined as key macrophage‐associated modules. Core macrophage‐related genes (MRGs) were selected from these 12 modules based on module eigengenes and intramodular connectivity.

### 2.4. Pseudotime Analysis and Cell–Cell Communication Analysis

To investigate the dynamics of macrophage differentiation and the temporal expression patterns of prognostic genes, macrophages were reclustered and analyzed. Macrophages were classified into distinct subpopulations, including M0, M1, and M2 phenotypes. Pseudotime trajectory analysis was conducted using Monocle (v2.28.0) to reconstruct macrophage developmental trajectories. Cells were ordered along a continuous differentiation path using the DDRTree algorithm, allowing for the identification of dynamic transcriptional changes across different differentiation states. Intercellular communication among macrophage subpopulations was inferred using the CellChat R package (v1.6.1). Macrophages were stratified based on *FCGR2B* expression, where *FCGR2B* ^+^ TAMs were defined as cells with detectable *FCGR2B* expression, and *FCGR2B* ^-^ TAMs were defined as cells with zero *FCGR2B* expression.

### 2.5. Identification of Differentially Expressed Genes (DEGs) and Candidate Genes

DEGs were identified in three contexts: scRNA‐DEGs (macrophages between HGSOC and normal samples), HGSOC‐DEGs (HGSOC vs. normal ovarian tissues in TCGA), and *FCGR2B*‐DEGs (*FCGR2B*‐high‐ and low‐expression HGSOC groups, stratified by the optimal cutoff, determined by the surv_cutpoint function in the survminer). Genes with |log2 fold change| > 0.5 and adjusted *p* < 0.05 were considered significantly differentially expressed. The intersection of scRNA‐DEGs, HGSOC‐DEGs, *FCGR2B*‐DEGs, MRGs, and FRGs was performed to identify core candidate genes.

### 2.6. Protein–Protein Interaction (PPI) Network Analysis of Candidate Genes

PPI network of candidate genes was constructed using STRING (v12.0, *Homo sapiens*, interaction score ≥ 0.15).

### 2.7. Prognostic Model Construction and Validation

Univariate Cox regression (survival v3.7‐0) was used to evaluate the association between candidate gene expression and OS in HGSOC. Genes with HR ≠ 1 and *p* < 0.05, satisfying proportional hazards assumptions (cox.zph, *p* > 0.05), were selected.

Random survival forest (RSF) (randomForestSRC v3.2.3; ntree = 100, mtry = 4, importance =  "permute") was used to rank variable importance and construct a prognostic model based on PH‐tested genes. Model performance was evaluated by the Kaplan–Meier survival analysis, log‐rank tests, and time‐dependent ROC curves, and validated in GSE102073. Patients were stratified into high‐ and low‐risk groups based on the optimal cutoff of RSF‐derived risk scores, determined using the survminer R package. To evaluate whether the risk score serves as an independent prognostic factor, a multivariable Cox proportional hazards regression analysis was performed. The continuous risk score was entered into the model together with key clinicopathological variables, including age and FIGO stage (HR ≠ 1 and *p* < 0.05). Additionally, for baseline clinical feature comparisons between high‐risk and low‐risk groups, chi‐square tests were used for categorical variables (*p* < 0.05), such as race and tumor stage, and the Mann–Whitney *U* test was applied for continuous variables like age (*p* < 0.05).

### 2.8. Gene Set Enrichment Analysis (GSEA) and Gene Set Variation Analysis (GSVA) Analysis

GSEA was performed between high‐ and low‐risk groups using clusterProfiler with MSigDB c5.go.v7.4.symbols gene sets; pathways with |normalized enrichment score (NES)| > 1, *p* < 0.05, and false discovery rate (FDR) < 0.25 were deemed significant. Additionally, GSEA was performed for individual prognostic genes by ranking all genes according to their Spearman correlation with each prognostic gene.

The ferroptosis‐related gene set WP_FERROPTOSIS was obtained from the Molecular Signatures Database (MSigDB) (http://www.gsea-msigdb.org/gsea/msigdb/index.jsp). GSVA was conducted using the GSVA R package (v1.53.28) to estimate ferroptosis pathway activity at the single‐cell level. GSVA scores were calculated for individual macrophages and subsequently compared between *FCGR2B* ^+^ and *FCGR2B* ^-^ TAMs subsets.

### 2.9. Immune Cell Infiltration Analysis

CIBERSORT (v1.03) was used to estimate the relative abundance of macrophage subtypes from TCGA HGSOC training cohorts, with 1000 permutations. Immune checkpoint gene (CD274, LAG3, LGALS9, HAVCR2, PDCD1, PDCD1LG2, and TIGIT) expression was compared. Differences between high‐ and low‐risk groups were assessed by the Wilcoxon rank‐sum test (adjusted *p* < 0.05).

### 2.10. Single‐Cell Regulatory Network Inference and Clustering (SCENIC) Analysis

SCENIC (v1.3.1) was applied to macrophage clusters to infer transcription factor–target regulons. Genes expressed in ≥ 3% of cells with counts > 1 were retained. GENIE3 was used for coexpression network inference, RcisTarget with hg19 motif databases for motif enrichment, and AUCell was used to calculate regulon activity.

### 2.11. Copy Number Variation (CNV) and Mutational Landscape Analyses

CNV profiles of the prognostic genes were obtained from the TCGA database. The proportions of gene amplifications and deletions were calculated, and Spearman correlation analysis was performed to assess the association between CNV status and gene expression. CNV correlations were visualized using the R package psych (v2.4.6.26). A more in‐depth analysis was conducted to compare the genetic differences between the two risk groups. The maftools (v 2.16.0) were used to analyze mutation data from patients in the two risk groups, and the Top 15 most frequently mutated genes were visualized utilizing waterfall plots. Subsequently, the distribution of somatic mutation types of prognostic genes was characterized, and the differences in tumor mutational burden (TMB) between the two groups were compared using the Wilcoxon rank‐sum test (*p* < 0.05).

### 2.12. Identification of Potential Therapeutic Compounds and Molecular Docking Analysis

Potential therapeutic compounds for HGSOC were predicted using the Enrichr platform with the Drug Signatures Database (DsigDB) based on prognostic genes. Drug–gene enrichment analysis was performed to identify compounds whose known targets or transcriptional signatures were significantly associated with the prognostic genes. Candidate drug–gene interaction networks were visualized using the ggraph R package (v2.2.2). Drug sensitivity was further evaluated using data from the Genomics of Drug Sensitivity in Cancer (GDSC) database. The pRRophetic package was applied to estimate IC50 values and IC50 values for chemotherapeutic agents were compared using the Wilcoxon rank‐sum test between two groups (adjusted *p* < 0.05).

3D protein structures were retrieved from PDB and prepared in PDBQT format using PyMOL v3.1.4 and AutoDockTools v1.5.7. Core compounds were converted from SMILES to 3D PDBQT using ChemBio2D v3.14 and AutoDockTools. Docking was performed with AutoDock Vina v1.2.x (binding energy ≤ −5.0 kcal/mol), and results were visualized in PyMOL and Discovery Studio v19.1.0.

### 2.13. Statistical Analysis

All statistical analyses and graph generation were performed using R software (Version 4.3.3). Results were visualized using ggplot2 (v3.5.1) and pheatmap (v1.0.12), unless otherwise specified. Chi‐squared test, Mann–Whitney test, Student′s *t*‐test, and Wilcoxon test were employed when appropriate to determine statistical differences between risk groups. *p* < 0.05 was considered statistically significant (∗*p* < 0.05, ∗∗*p* < 0.01, ∗∗∗*p* < 0.001, ∗∗∗∗*p* < 0.0001).

## 3. Results

### 3.1. *FCGR2B* ^+^ TAMs Are Prognostic and Enriched in the HGSOC TME

We first evaluated the prognostic relevance of *FCGR2B* in HGSOC by analyzing OS in the TCGA training cohort. Kaplan–Meier analysis revealed a time‐dependent association: High *FCGR2B* expression correlated with poorer early‐stage OS, whereas low expression was linked to worse long‐term survival (log − rank *p* = 0.03; Figure 1a).

Figure 1
*FCGR2B* is associated with prognosis and is predominantly expressed by macrophages in HGSOC. (a) Kaplan–Meier analysis of overall survival in HGSOC patients stratified by high and low *FCGR2B* expression using an optimal cutoff in the TCGA training cohort (*n* = 399). Survival differences were assessed by the log‐rank test. (b) Single‐cell RNA‐seq analysis identifying major cell populations in HGSOC, including fibroblasts, T cells, macrophages, epithelial cells, proliferating T cells, endothelial cells, B cells, and smooth muscle cells. (c) UMAP visualization showing *FCGR2B* expression across single‐cell populations, revealing enrichment of *FCGR2B* in macrophages. (d) Half‐violin plots showing *FCGR2B* expression in distinct cell types from HGSOC and normal ovarian tissues in the GSE184880 dataset. Statistical significance was evaluated using the Wilcoxon rank‐sum test. Statistical significance is indicated as follows:  ^∗^ *p* < 0.05,  ^∗∗^ *p* < 0.01,  ^∗∗∗^ *p* < 0.001; N.S., nonsignificant.

**Figure:**
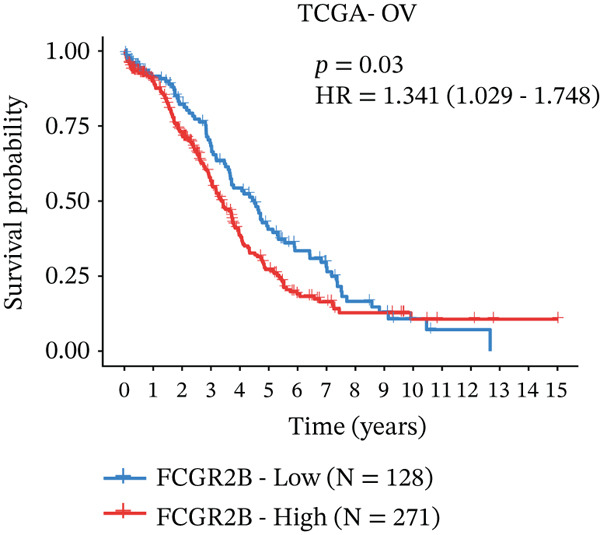
(a)

**Figure:**
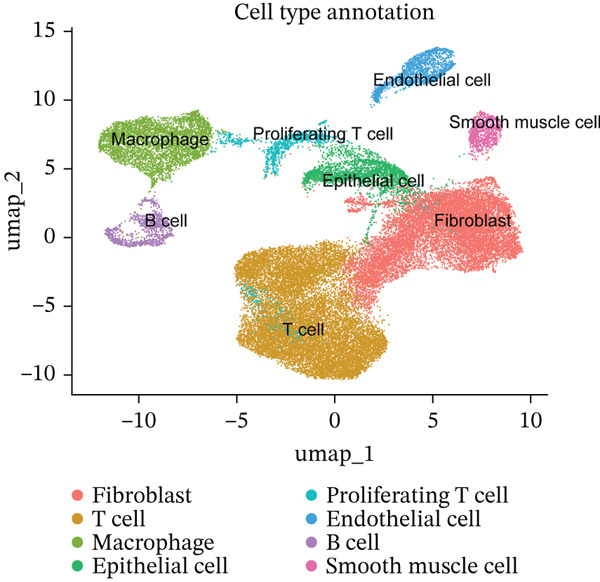
(b)

**Figure:**
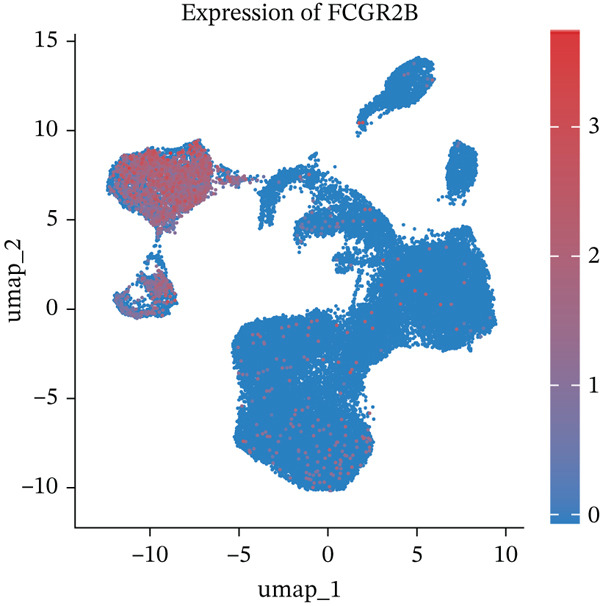
(c)

**Figure:**
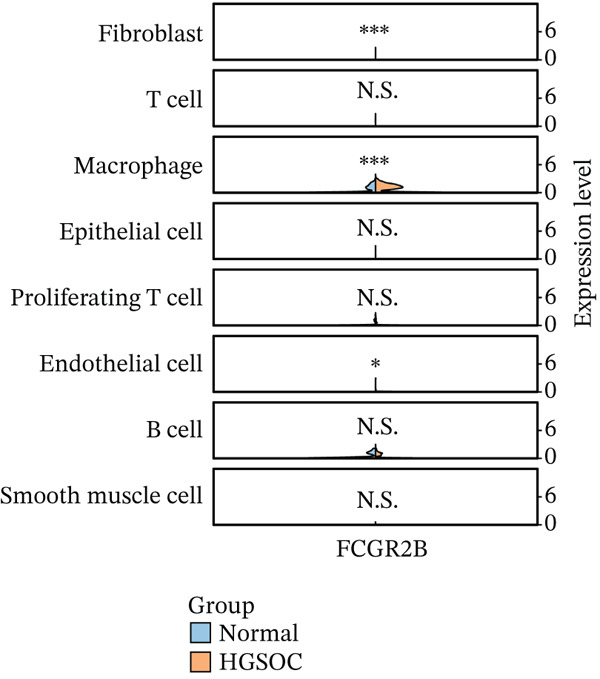
(d)

To explore the cellular basis of this association, we analyzed *FCGR2B* expression at single‐cell resolution using data from the GSE184880 dataset. We identified eight major cell types (38,949 filtered cells), including fibroblasts, T cells, macrophages, epithelial cells, endothelial cells, B cells, proliferating T cells, and smooth muscle cells (Figure 1b). Data underwent standard quality control and batch correction (Figures S1a, S1b, S1c, and S1d). Compared with normal ovarian tissues, HGSOC samples exhibited significant alterations in cellular composition, including increases in T cells, macrophages, epithelial cells, proliferating T cells, and B cells, alongside decreases in fibroblasts, endothelial cells, and smooth muscle cells (Figure S1e,f). *FCGR2B* expression was predominantly enriched in macrophages and was significantly higher in HGSOC‐derived macrophages compared with those from normal tissues (Wilcoxon rank‐sum test, *p* < 0.001; Figure 1c,d). These results establish *FCGR2B* as a marker predominantly expressed in macrophages, providing a cellular basis for its prognostic relevance in HGSOC.

### 3.2. *FCGR2B* ^+^ TAMs Exhibit Altered Intercellular Communication and Elevated Ferroptosis Activity in HGSOC

We next characterized the intercellular signaling networks of *FCGR2B* ^+^ TAMs in both normal and HGSOC tissues. In normal tissues, *FCGR2B* ^+^ TAMs frequently interacted with T cells, fibroblasts, endothelial cells, and *FCGR2B* ^−^ TAMs, displaying the strongest outgoing signals to fibroblasts (Figure 2a,b, left). Key ligand–receptor pairs, such as NAMPT‐INSR and LGALS9‐CD45, mediated these interactions (Figure S2). In HGSOC, this communication profile was markedly altered. Interactions with fibroblasts were diminished, whereas communications with T cells were selectively enhanced (Figure 2a,b, right). Ligand–receptor analysis revealed that *FCGR2B* ^+^ TAMs, as signaling sources, engaged T cells via HLA‐A/B‐CD8A/B pairs, and as targets, received strong signals from endothelial cells via APP‐CD74 (Figure 2c). In addition, *FCGR2B* ^+^ TAMs exhibited significantly higher ferroptosis pathway activity compared with their *FCGR2B* ^−^ counterparts (*p* < 0.05; Figure 2d), indicating a subset‐specific activation of ferroptosis within the TME. Collectively, these results demonstrate that *FCGR2B* ^+^ TAMs in HGSOC undergo coordinated rewiring of their communication networks and ferroptosis activity, underscoring a specific focus on T‐cell interactions within the TME.

Figure 2
*FCGR2B* ^+^ TAMs exhibit tumor‐specific intercellular communication features and altered ferroptosis signaling in HGSOC. (a) Comparison of intercellular communication networks based on interaction number in the normal ovaries (left) and HGSOC (right). (b) Comparison of intercellular communication networks based on interaction strength in the normal ovaries (left) and HGSOC (right). (c) Bubble plot showing key ligand–receptor interactions involving *FCGR2B* ^+^ and *FCGR2B* ^-^ TAMs as signaling sources (left) or targets (right) in HGSOC. The color of the bubbles represents the interaction strength, and the size indicates the statistical significance (*p* value) of the interactions. (d) Boxplots of GSVA‐derived ferroptosis pathway scores in *FCGR2B* ^+^ versus *FCGR2B* ^-^ TAMs. Statistical significance was assessed using the Wilcoxon rank‐sum test ( ^∗^ *p* < 0.05,  ^∗∗^ *p* < 0.01,  ^∗∗∗^ *p* < 0.001; N.S., nonsignificant).

**Figure:**
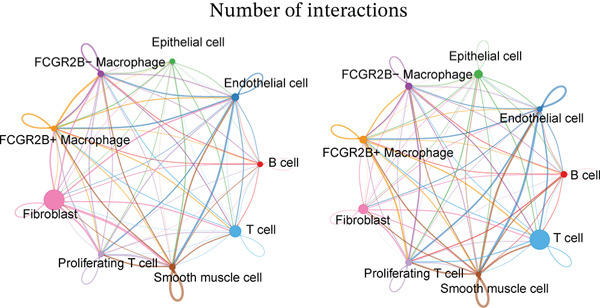
(a)

**Figure:**
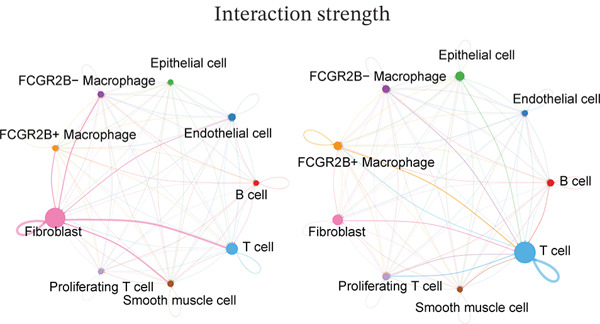
(b)

**Figure:**
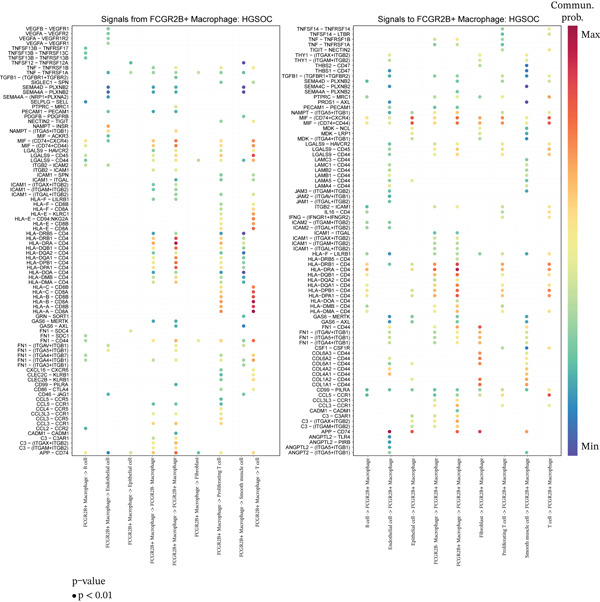
(c)

**Figure:**
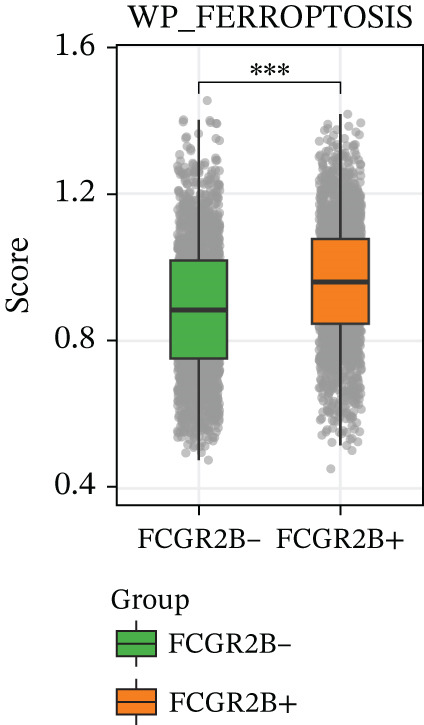
(d)

### 3.3. Identification of Candidate Genes and PPI Networks Associated With *FCGR2B* ^+^ TAMs

Building on the functional characterization of *FCGR2B* ^+^ TAMs, differential expression analysis identified HGSOC‐associated DEGs, *FCGR2B*‐associated DEGs, and scRNA‐DEGs, which were then integrated to select candidate genes (Figures 3a, 3b, and 3c and Tables S2, S3, and S4; details in Method). To further characterize macrophage‐associated gene networks, hdWGCNA was applied, identifying 14 gene modules (excluding gray) after metacell aggregation and scale‐free network construction (Figures S3a, S3b, S3c, S3d, and S3e). Several modules showed enrichment in macrophage subpopulations, from which 1572 MRGs were selected for downstream analyses (Figure 3d,e and Table S5).

Figure 3Identification and functional characterization of *FCGR2B*‐associated candidate genes. (a–c) Volcano plots showing differential expression analyses across different datasets and stratifications. The *x*‐axis represents log2 fold change, and the *y*‐axis represents –y (adjusted *p* value). Each dot represents one gene. Red dots indicate significantly upregulated genes, blue dots indicate downregulated genes, and gray dots indicate genes without significant differential expression. (a) HGSOC‐associated DEGs: Differentially expressed genes (DEGs) between HGSOC and normal ovarian samples in the TCGA cohort. (b) *FCGR2B*‐associated DEGs: DEGs between high and low *FCGR2B* expression groups using the optimized cutoff within HGSOC samples. (c) scRNA‐DEGs: Macrophage‐specific DEGs between HGSOC and normal samples derived from scRNA‐seq data (GSE184880). (d) Weighted gene coexpression network constructed using hdWGCNA. Each node represents a gene, and labeled nodes indicate the Top 2 hub genes with the highest intramodular connectivity in each module. Edges represent gene–gene correlations, with only 10% of significant associations randomly sampled for visualization. Nonspecific gray edges were removed for clarity. (e) Bubble plot showing module–cell type associations based on the expression of module core genes. The *x*‐axis indicates cell types, and the *y*‐axis indicates gene coexpression modules. Bubble size represents the proportion of cells expressing module core genes, whereas color intensity reflects the average expression level. (f) Venn diagram illustrating the overlap among macrophage‐specific DEGs from scRNA‐DEGs, HGSOC‐associated DEGs, *FCGR2B*‐associated DEGs, macrophage‐related genes (MRGs) identified by hdWGCNA, and ferroptosis‐related genes. (g) Protein–protein interaction (PPI) network of 26 candidate genes constructed using the STRING database (interaction score ≥ 0.15). Edges represent PPIs. Node color (red to blue) reflects the number of interactions (degree), with red indicating higher connectivity. Edge color intensity (yellow gradient) represents interaction confidence, with darker colors indicating higher confidence.

**Figure:**
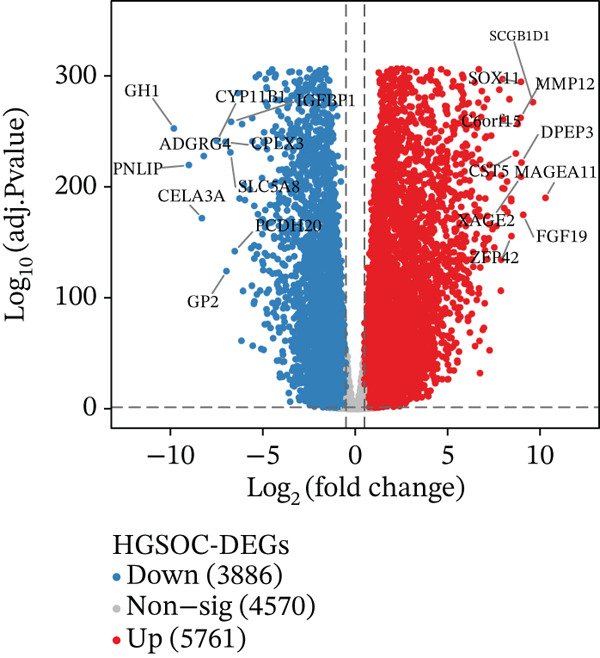
(a)

**Figure:**
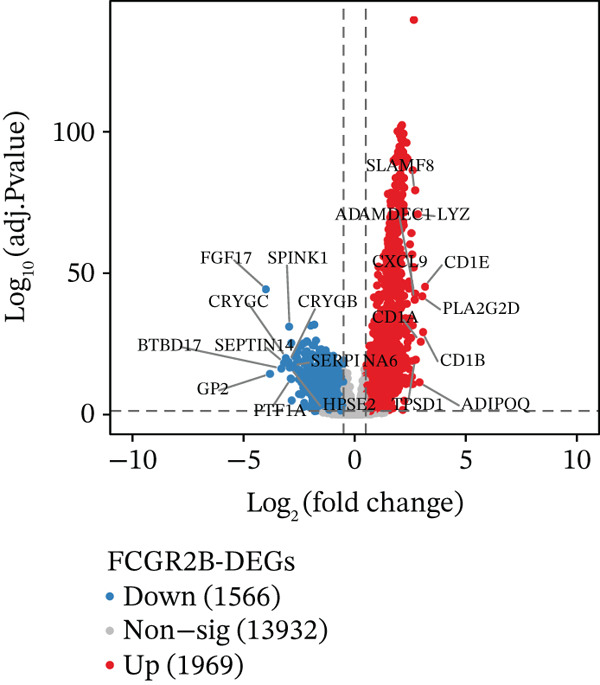
(b)

**Figure:**
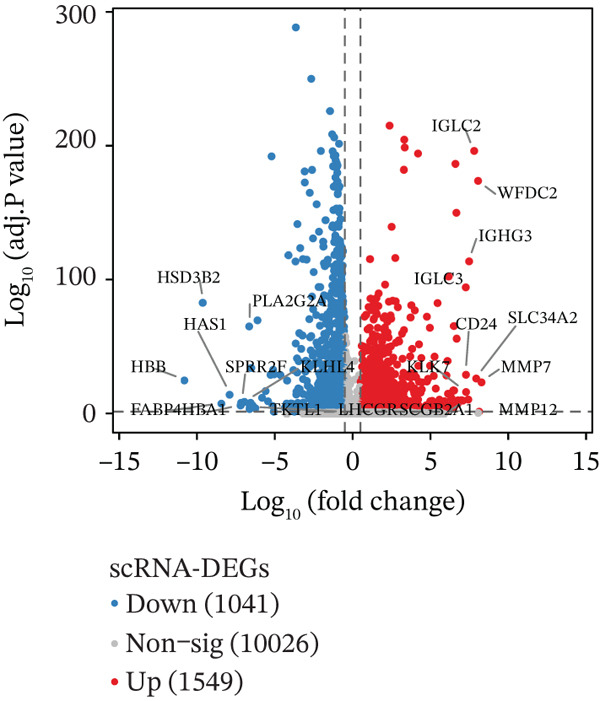
(c)

**Figure:**
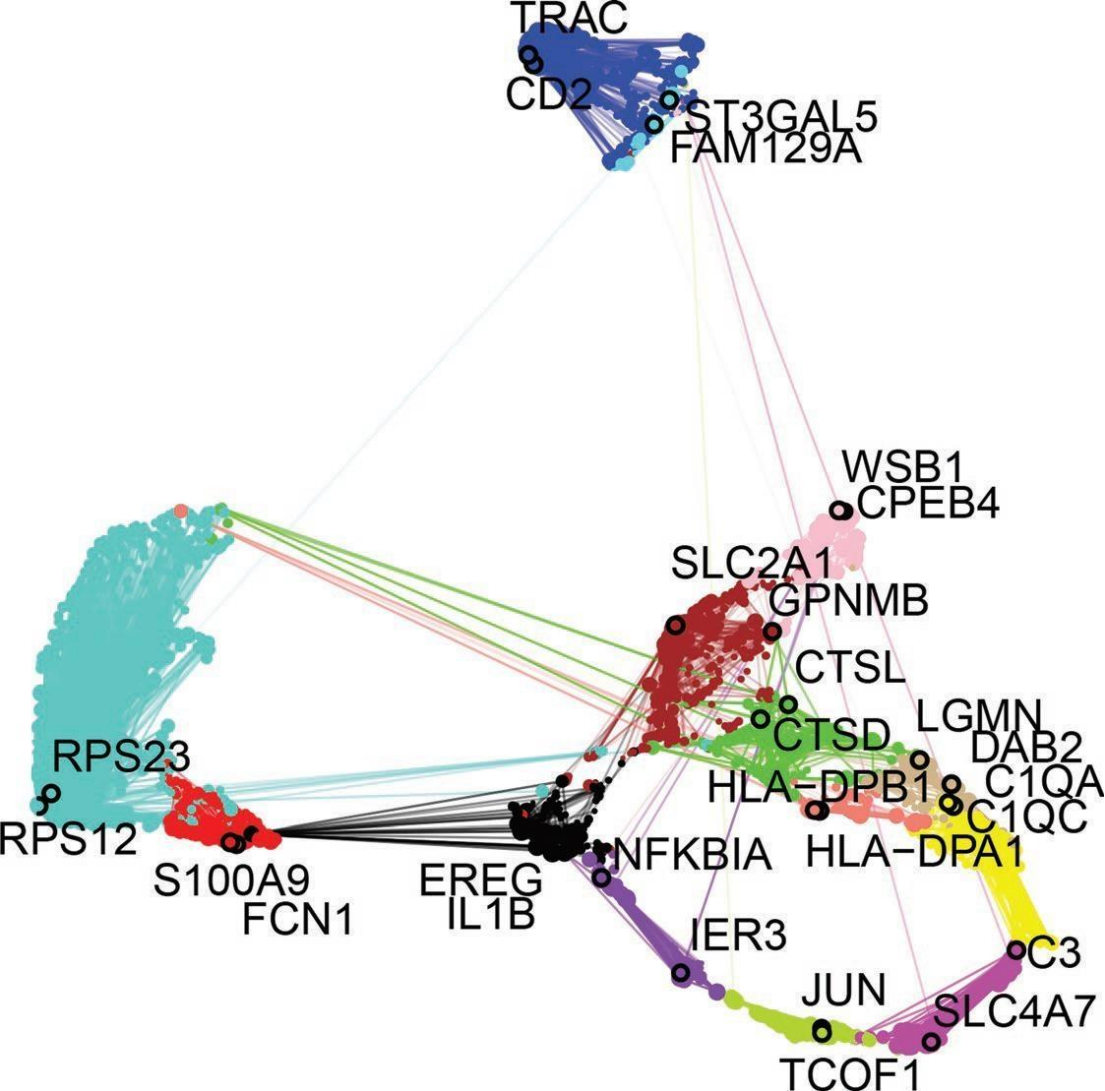
(d)

**Figure:**
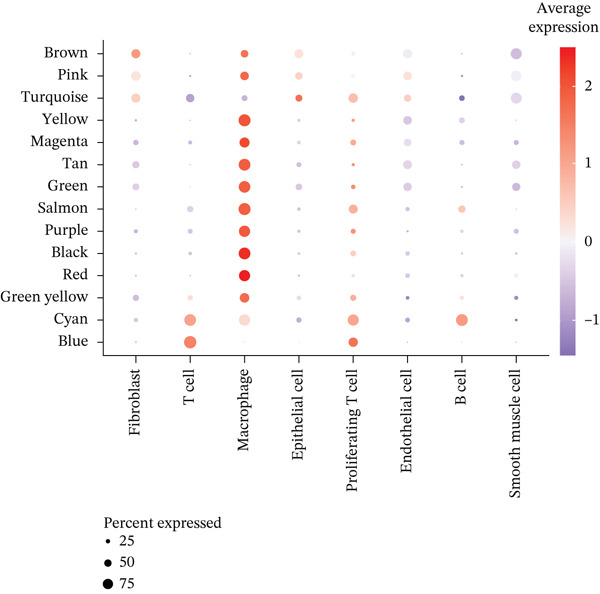
(e)

**Figure:**
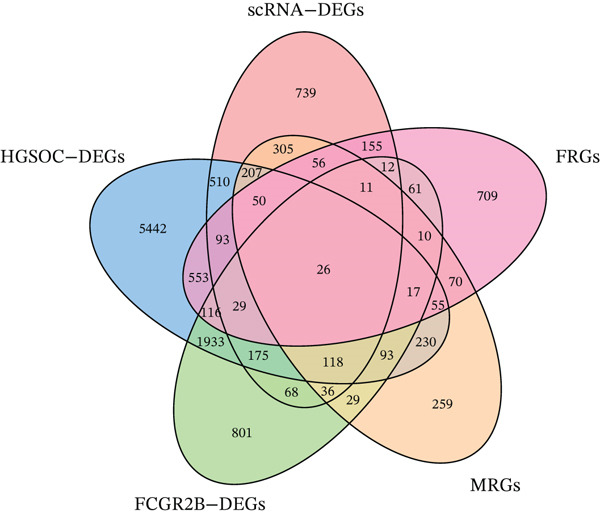
(f)

**Figure:**
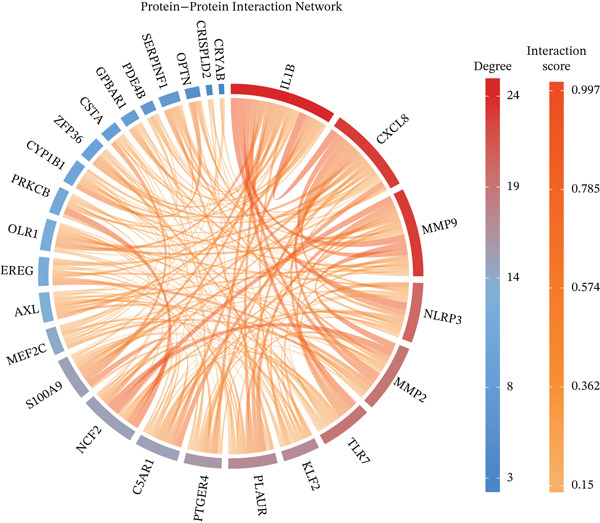
(g)

Integrating HGSOC‐associated DEGs, *FCGR2B*‐associated DEGs, scRNA‐DEGs, MRGs, and a curated ferroptosis gene set (Table S6) yielded 26 candidate genes (Figure 3f). PPI network analysis revealed 23 interconnected genes, with IL1B, CXCL8, and MMP9 identified as hub genes based on network connectivity (Figure 3g). Together, these analyses define a set of candidate genes with potential relevance to macrophage biology and ferroptosis in HGSOC.

### 3.4. Derivation and Validation of a Prognostic Model Based on Candidate Macrophage‐Ferroptosis Genes

To evaluate the prognostic utility of the candidate genes, univariate Cox regression identified four genes (*CRYAB*, *PLAUR*, *EREG*, and *C5AR1*) significantly associated with shorter OS (hazard ratio [HR] > 1, *p* < 0.05; Figure 4a and Table S7). The proportional hazards assumption was satisfied for all four genes based on Schoenfeld residual tests (*p* > 0.05; Figure S4a). We then constructed an RSF model incorporating these genes. Variable importance ranking within the RSF highlighted *CRYAB*, *C5AR1*, *EREG*, and *PLAUR* as key contributors to risk prediction (Figure 4b). Using the RSF‐derived risk scores, we stratified patients in the training cohort into high‐ and low‐risk groups. High‐risk patients exhibited significantly shorter OS compared with low‐risk patients (Figure 4c,d; *p* < 0.0001). The model demonstrated strong predictive performance, with time‐dependent area under the curve (AUC) values of 0.690, 0.769, and 0.841 at 1, 3, and 5 years, respectively (Figure 4e). In the independent validation cohort, the risk stratification remained robust: High‐risk patients again showed higher mortality (*p* = 0.005) and shorter OS, with 1‐, 3‐, and 5‐year AUCs of 0.721, 0.638, and 0.605, respectively (Figures 4f, 4g, and 4h). This confirms the model′s generalizability. Together, these results demonstrate that the four‐gene RSF signature (*CRYAB*, *PLAUR*, *EREG*, and *C5AR1*) effectively stratifies HGSOC patients by prognosis, providing a reliable tool for survival prediction. Multivariable Cox regression analysis demonstrated that the risk score remained a significant independent prognostic factor after adjustment for age and FIGO stage (HR = 1.026, 95% CI: 1.021–1.030, *p* < 0.001) (Figure S4b), indicating that its prognostic value was not confounded by major clinicopathological variables. In addition, no significant differences in baseline clinical characteristics, such as age, race, or tumor stage, were observed between the high‐risk and low‐risk groups (*p* > 0.05 for all comparisons) (Table S8), further supporting the independence of the RSF‐derived risk score.

Figure 4Construction and validation of a prognostic risk model based on *FCGR2B*‐associated candidate genes. (a) Forest plot of univariate Cox regression analysis for candidate genes in the training cohort. Hazard ratios (HR) and 95% confidence intervals are shown. Genes with HR values below 1 and above 1 are shown in yellow and blue, respectively, for visualization. (b) Construction of a random survival forest (RSF) model in the training cohort. The left panel shows the model error curve across 50 survival trees, indicating stable model performance. The right panel shows variable importance for the four prognostic candidate genes. (c–e) Distribution of risk scores (top) and corresponding survival status (bottom), Kaplan–Meier curves comparing overall survival between high‐ and low‐risk groups, and time‐dependent ROC curves for predicting 1‐, 3‐, and 5‐year OS in the training cohort. (f–h) Corresponding analyses in the validation cohort.

**Figure:**
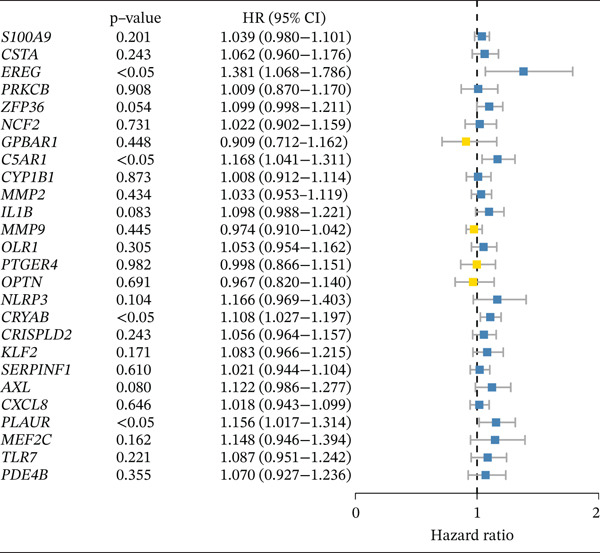
(a)

**Figure:**
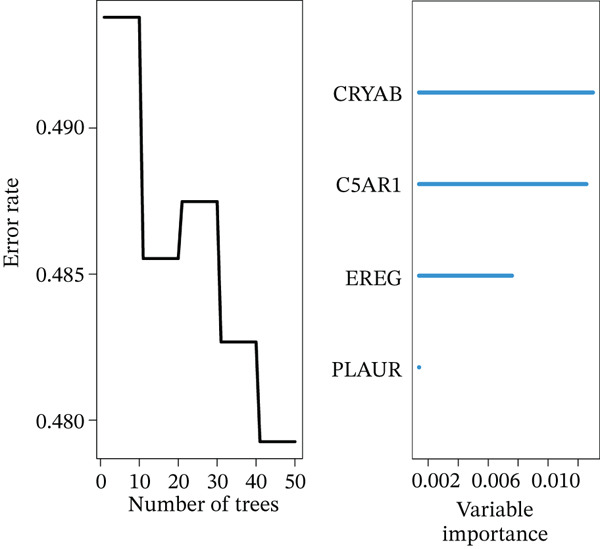
(b)

**Figure:**
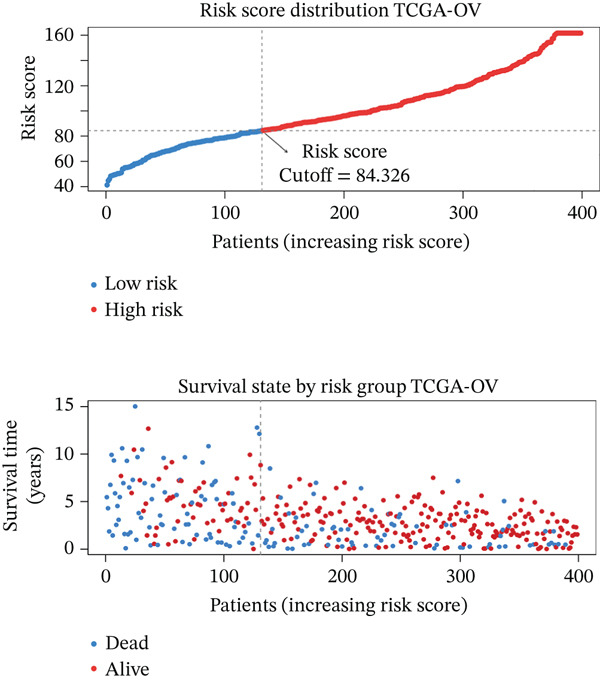
(c)

**Figure:**
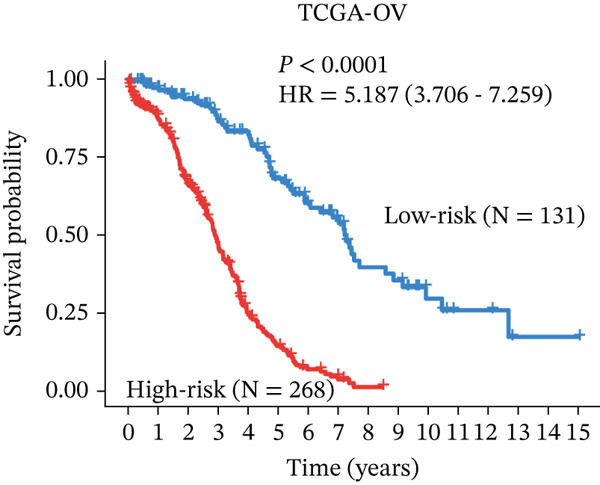
(d)

**Figure:**
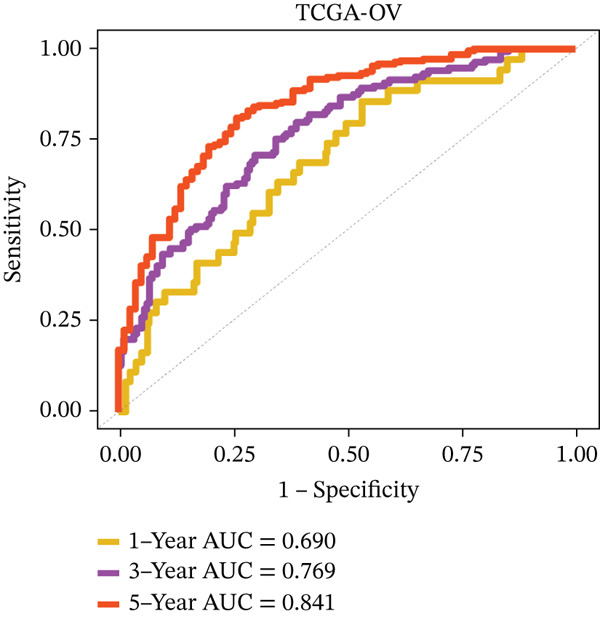
(e)

**Figure:**
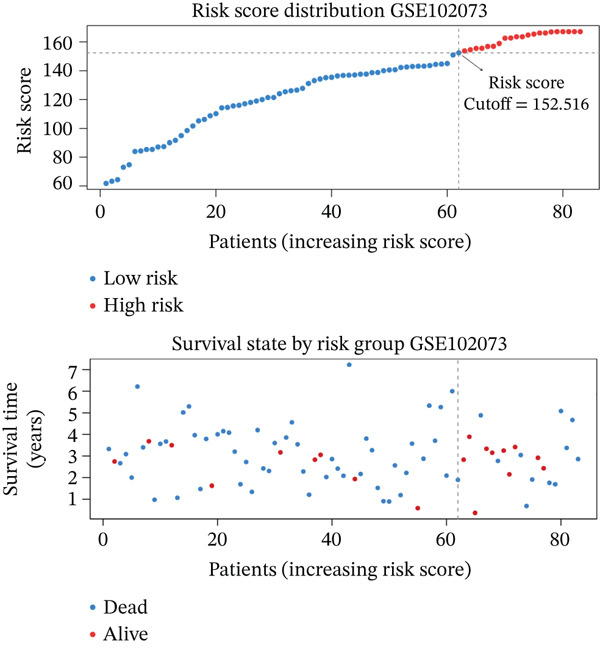
(f)

**Figure:**
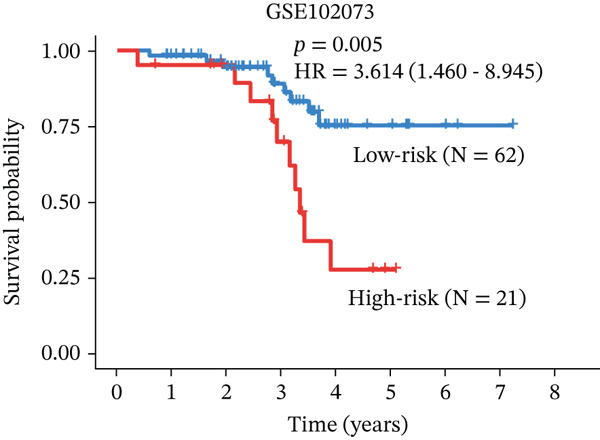
(g)

**Figure:**
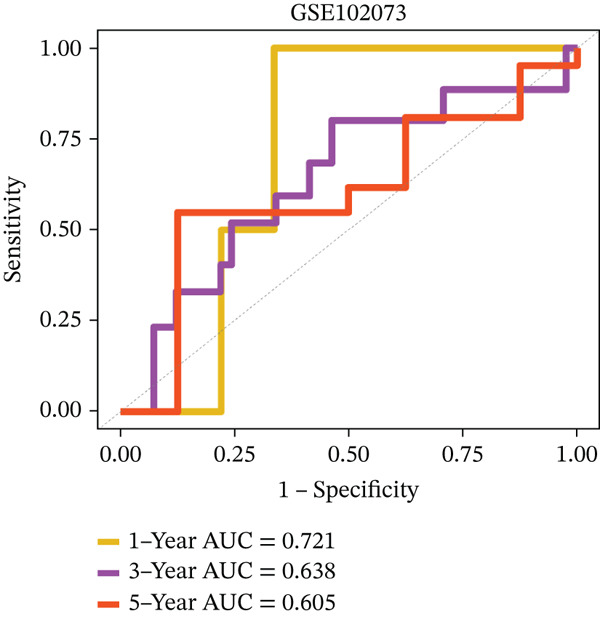
(h)

### 3.5. The High‐Risk Prognostic Signature Reflects an Immunosuppressive and Ferroptosis‐Associated Microenvironment

To characterize the immune microenvironment associated with the prognostic signature, we used CIBERSORT to quantify macrophage subtypes in high‐ and low‐risk patients (Figure 5a). The high‐risk group exhibited a significant increase in M2 macrophages, suggesting an immunosuppressive TME. Analysis of immune checkpoint genes revealed their widespread upregulation in high‐risk patients, with HAVCR2 (TIM‐3) and PDCD1LG2 (PD‐L2) being notably elevated (Figure 5b). GSEA further revealed enrichment of immune‐related pathways in the high‐risk group, including leukocyte migration and cytokine production regulation (Figure 5c), suggesting enhanced immune cell recruitment and activity. These enriched pathways overlapped substantially with the top biological processes shared by the four prognostic genes (Figure 5d and Table S9), highlighting the genes′ central role in regulating immune processes such as leukocyte migration and T‐cell activation. Furthermore, analysis of iron metabolism–related pathways indicated their potential link to ferroptosis (Figures 5e, 5f, 5g, and 5h). Collectively, these analyses indicate that the high‐risk prognostic signature is associated with an immunosuppressive TME and altered iron metabolism/ferroptosis pathways.

Figure 5Immune microenvironment characterization in high‐ and low‐risk HGSOC patients. (a) Differential infiltration of macrophage subtypes between high‐ and low‐risk HGSOC patients estimated by CIBERSORT, with significant differences indicated by the Wilcoxon rank‐sum test (*p* < 0.05). (b) Expression levels of representative immune checkpoint genes in high‐ and low‐risk groups. (c) Gene set enrichment analysis (GSEA) showing pathways significantly enriched between high‐ and low‐risk patients. (d) Heatmap showing normalized enrichment scores (NES) of pathways associated with the four prognostic genes (*CRYAB*, *PLAUR*, *EREG*, and *C5AR1*). Each row represents a pathway, and each column represents a gene. Higher NES values indicate stronger enrichment of the pathway. (e–h) GSEA results for iron metabolism‐related pathways corresponding to each of the four prognostic genes, highlighting pathway enrichment patterns in high‐ and low‐risk patients. For (a) and (b), red boxes indicate the high‐risk group and blue boxes indicate the low‐risk group. Statistical significance is indicated as follows:  ^∗^ *p* < 0.05,  ^∗∗^ *p* < 0.01,  ^∗∗∗^ *p* < 0.001; N.S., nonsignificant.

**Figure:**
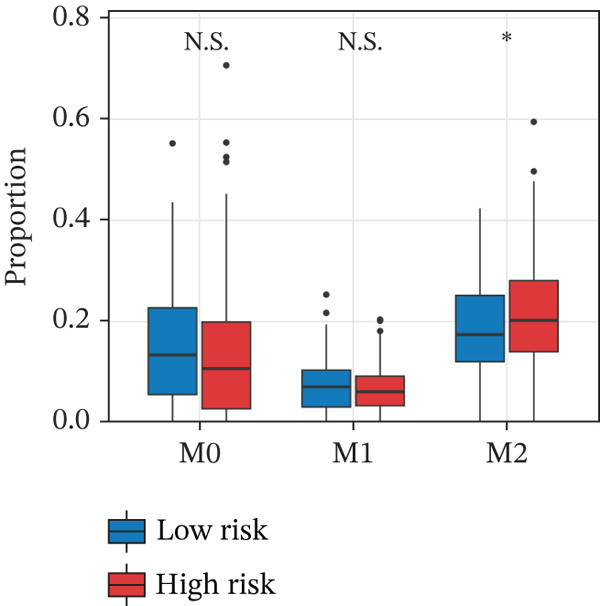
(a)

**Figure:**
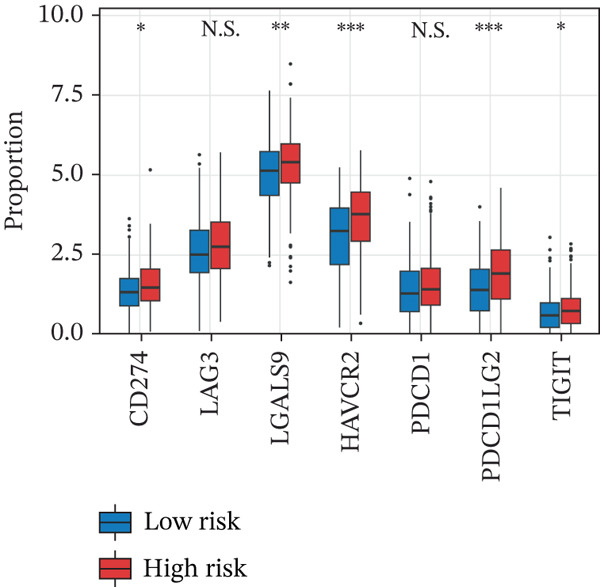
(b)

**Figure:**
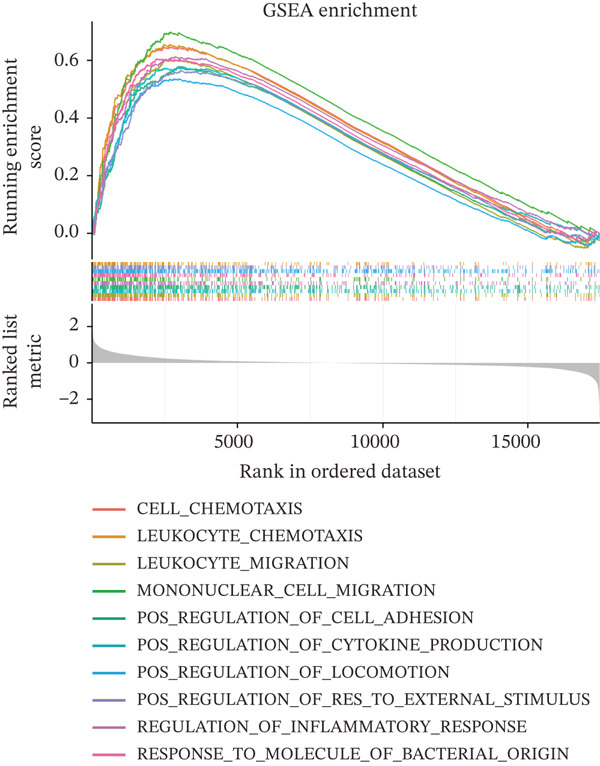
(c)

**Figure:**
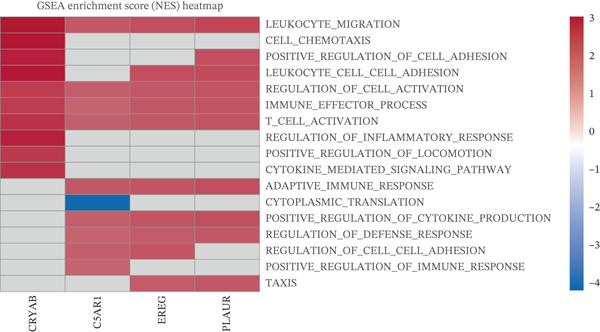
(d)

**Figure:**
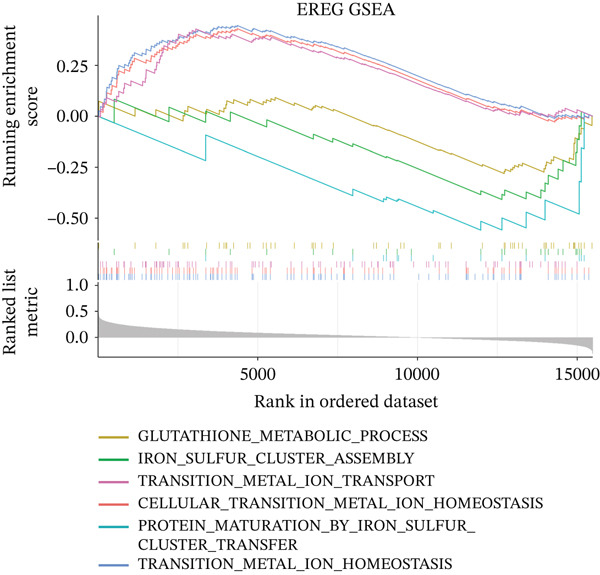
(e)

**Figure:**
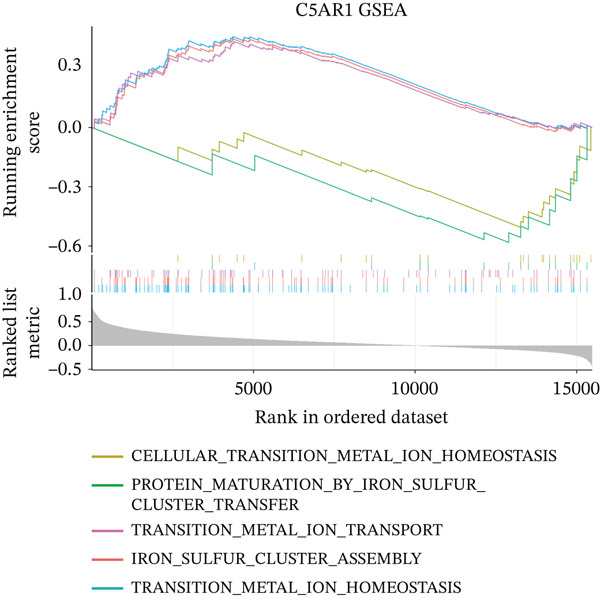
(f)

**Figure:**
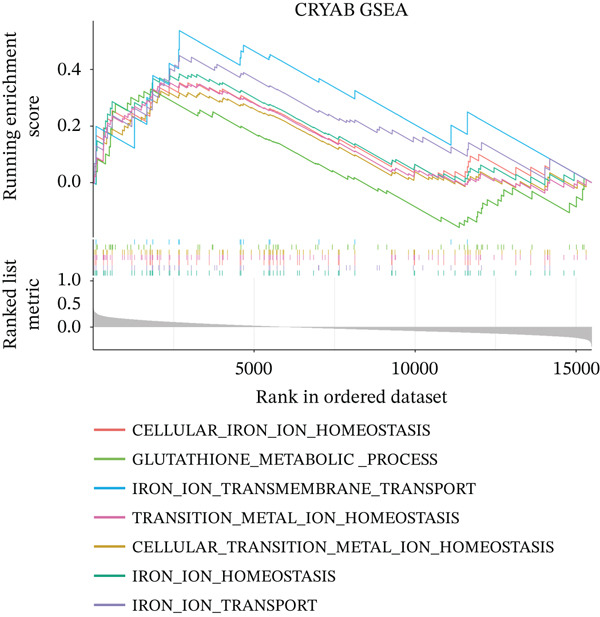
(g)

**Figure:**
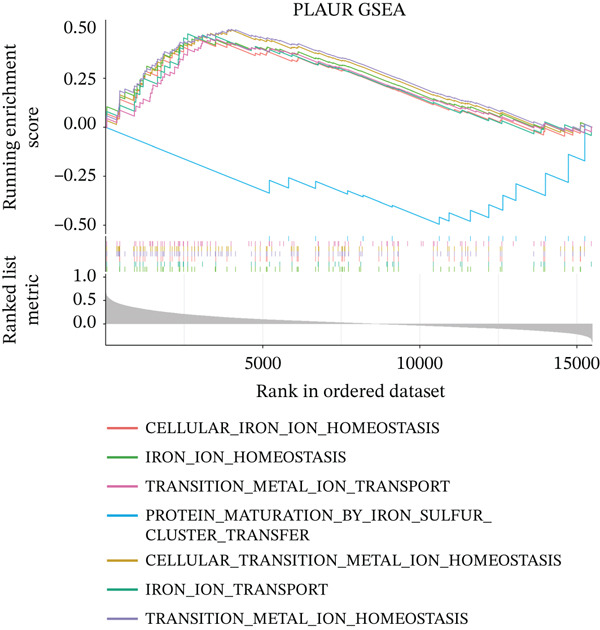
(h)

### 3.6. Prognostic Genes Exhibit Dynamic Expression During Macrophage Differentiation, Influenced by Transcription Factors and Copy Number Alterations

To investigate the dynamics of the prognostic genes during macrophage differentiation, we performed reclustering and trajectory inference on macrophages, revealing a continuous spectrum of transcriptional states (Figure 6a,b). The inferred pseudotime trajectory confirmed a continuous differentiation spectrum, with cells from all samples distributed across this continuum, supporting robust pseudotemporal ordering (Figures S5a, S5b, and S5c). The four prognostic genes exhibited distinct temporal patterns: *C5AR1* and *PLAUR* expression remained relatively stable, *CRYAB* increased in later pseudotime, and *EREG* decreased along the trajectory (Figure 6c), suggesting stage‐specific regulation. Regulatory network analysis identified key transcription factors (*e.g.*, CEBPB, SPI1, and IRF7) that potentially coordinate these expression programs in macrophages (Figure 6d). Furthermore, analysis of CNVs in TCGA cohorts revealed frequent amplifications of *CRYAB*, *PLAUR*, and *EREG* (Figure 6e). These amplifications were positively correlated with mRNA expression levels for *CRYAB*, *PLAUR*, and *EREG*, whereas *C5AR1* expression was independent of CNV status (Figure S6a). This suggests that CNV‐driven amplification contributes to the elevated expression of *CRYAB*, *PLAUR*, and *EREG*, potentially shaping TAM functional heterogeneity and impacting HGSOC prognosis. In summary, the prognostic genes display dynamic, stage‐specific expression during macrophage differentiation, regulated by key transcription factors. Additionally, CNV‐driven amplification contributes to the overexpression of *CRYAB*, *PLAUR*, and *EREG*, potentially influencing TAM phenotypes and patient prognosis. To gain deeper insights into genetic mutations between the two risk groups, somatic mutation profiles were analyzed. Both groups predominantly harbored missense mutations (Figure S6b,c). The most frequently mutated genes in the high‐risk group were TP53, TTN, and FLG2, whereas TP53, TTN, and CSMD3 were the top mutated genes in the low‐risk group, highlighting differential genomic landscapes associated with risk stratification. Missense mutations were observed only in *C5AR1* among the four prognostic genes (Figure S6d). TMB did not differ significantly between the high‐ and low‐risk groups (*p* > 0.05; Figure S6e), indicating that the prognostic stratification is independent of overall mutation load.

Figure 6Macrophage pseudotime trajectory and transcriptional landscape of prognostic genes in HGSOC. (a) UMAP visualization of macrophage subclusters in the GSE184880 dataset. Each point represents a single cell, colored according to its annotation. (b) Pseudotime trajectory of macrophages inferred using Monocle. Colors indicate basic cluster (left) and pseudotime progression (right). (c) Expression dynamics of prognostic genes (*C5AR1*, *CRYAB*, *EREG*, and *PLAUR*) along the pseudotime trajectory. The heatmap shows changes in gene expression across differentiation stages, with color intensity representing scaled expression levels. (d) SCENIC‐based transcriptional regulatory network analysis of macrophages. Heatmap depicts the regulon activity (AUC) across macrophage subclusters. (e) The distribution of copy number variation types (amplification, deletion, and normal) for *C5AR1*, *CRYAB*, *EREG*, and *PLAUR* is shown.

**Figure:**
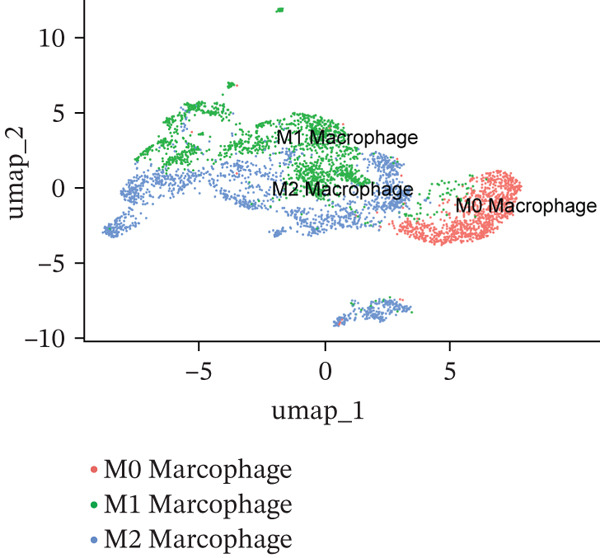
(a)

**Figure:**
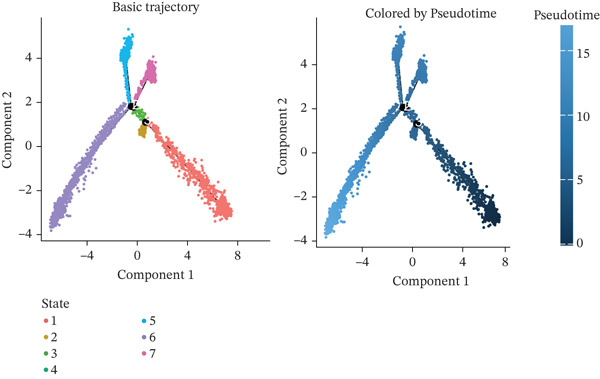
(b)

**Figure:**
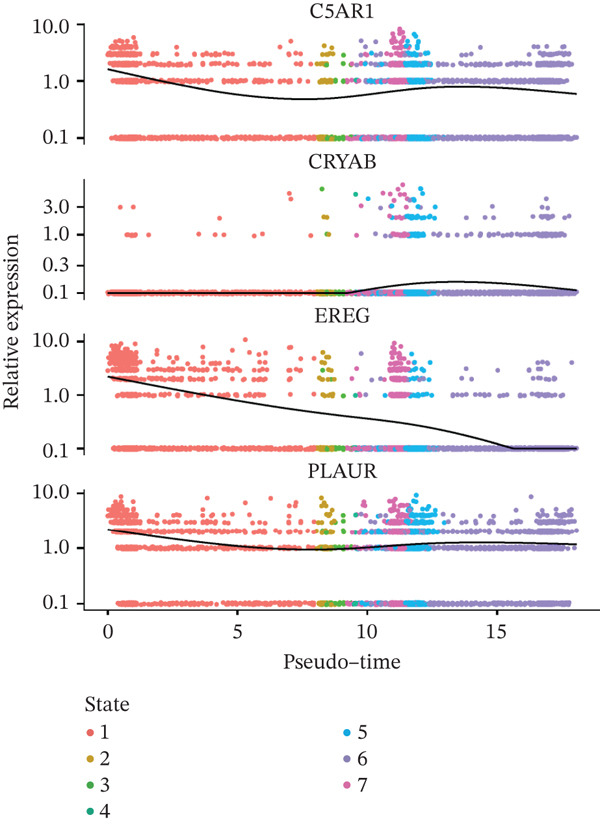
(c)

**Figure:**
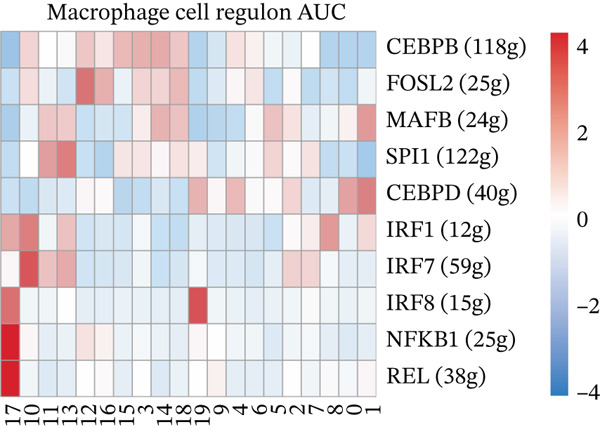
(d)

**Figure:**
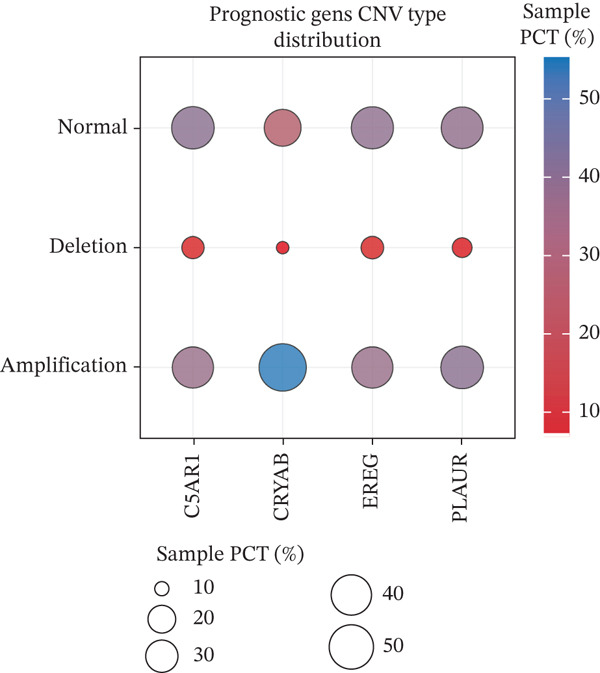
(e)

### 3.7. Prioritization of Candidate Therapeutic Compounds Via Drug‐Gene Interaction Analysis

To translate the prognostic findings into therapeutic insights, we performed molecular docking and drug–gene network analysis to identify compounds potentially targeting the four prognostic genes. Docking analysis predicted stable binding affinities (≤ −5.0 kcal/mol) between candidate drugs and the target proteins. The strongest predicted interactions were between *PLAUR* and 1,4‐chrysenequinone (−8.6 kcal/mol), and between *C5AR1* and estrone sulfate (−8.1 kcal/mol). Additionally, estrone sulfate and dexamethasone showed favorable binding affinities with *EREG* (−6.7 kcal/mol) and *CRYAB* (−6.3 kcal/mol), respectively (Figures 7a, 7b, 7c, and 7d and Table S10). Drug–gene network analysis visually confirmed these overlapping and specific interaction patterns, showing dexamethasone targeting three genes, and compounds like estrone sulfate and 9‐anthroic acid showing dual‐ or single‐gene specificity (Figure 7e and Table S11).

Figure 7Molecular docking and drug‐gene associations of four prognostic genes (*CRYAB*, *PLAUR*, *EREG*, and *C5AR1*) in HGSOC. (a–d) Molecular docking of prognostic genes with selected drugs: (a) *EREG* with estrone sulfate, (b) *C5AR1* with estrone sulfate, (c) *CRYAB* with dexamethasone, and (d) *PLAUR* with 1,4‐chrysenequinone. The predicted binding affinity (kcal/mol) for each interaction is indicated. (e) Network of predicted drug‐gene associations involving the four prognostic genes. Edges represent predicted interactions, with thickness proportional to interaction score. An adjusted *p* < 0.5 was used as a permissive threshold for exploratory analysis. (f) Predicted drug sensitivity (IC50) for the Top 10 candidate compounds in high‐ and low‐risk HGSOC patients. Boxplots indicate median and interquartile range; lower IC50 values correspond to higher predicted sensitivity. Statistical significance was assessed by the Wilcoxon rank‐sum test (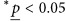, 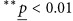, 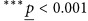; N.S., nonsignificant).

**Figure:**
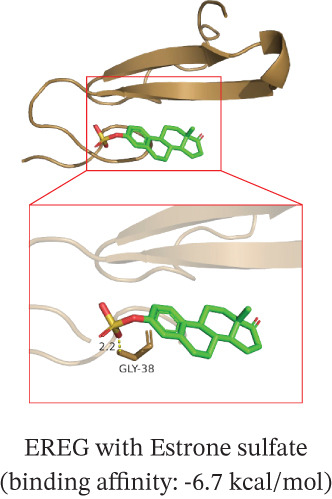
(a)

**Figure:**
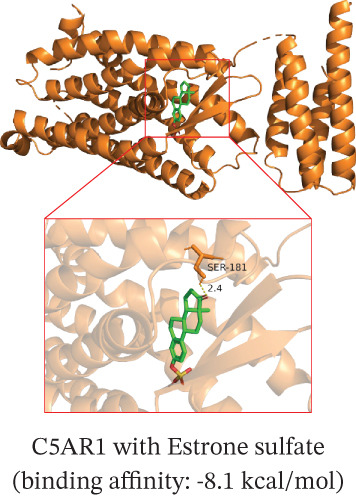
(b)

**Figure:**
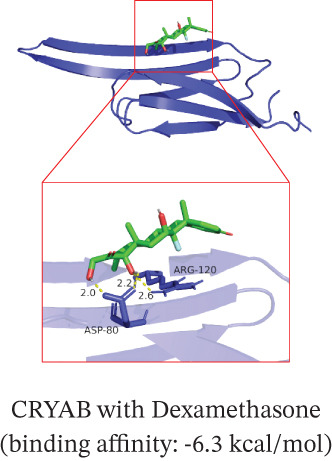
(c)

**Figure:**
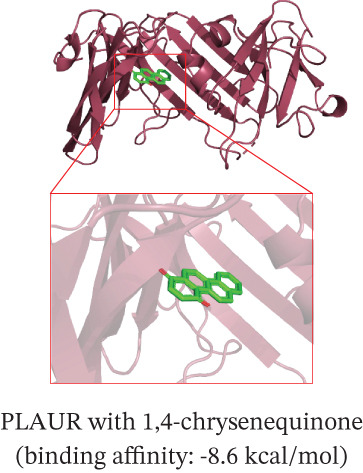
(d)

**Figure:**
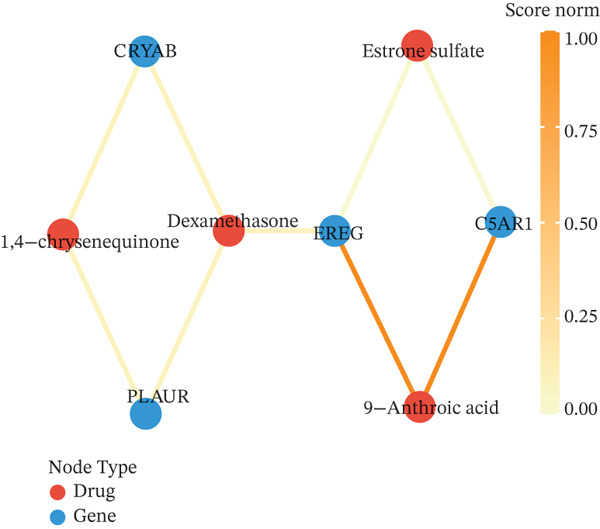
(e)

**Figure:**
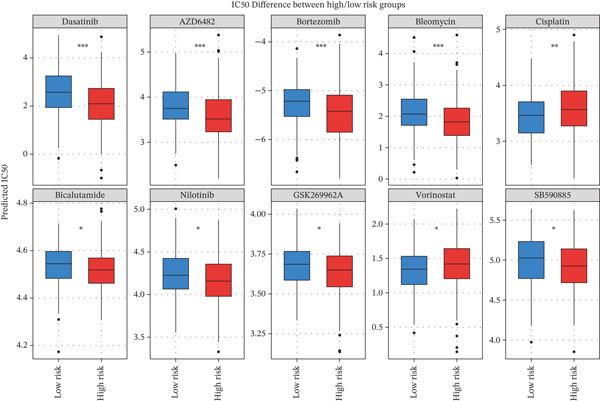
(f)

Analysis of predicted half‐maximal inhibitory concentrations (IC50) using the GDSC database revealed significant differences in drug sensitivity between the risk groups. Notably, high‐risk patients showed predicted sensitivity to targeted agents (*e.g.*, dasatinib) and proteasome inhibitors (*e.g.*, bortezomib), whereas low‐risk patients were predicted to be more sensitive to classical chemotherapy (*e.g.*, cisplatin; Wilcoxon rank‐sum test, *p* < 0.05; Figure 7f). This suggests the RSF signature could inform personalized therapeutic strategies.

## 4. Discussion

Previous studies have established that HGSOC is characterized by poor prognosis, substantial intratumoral heterogeneity, and a complex TME [22, 23]. Within this milieu, TAMs constitute a dominant immune population and critically contribute to immunosuppression, tumor progression, and resistance to therapy [24–26]. Furthermore, TAMs exhibit significant metabolic plasticity, where the reprogramming of nutrient pathways dictates their polarization, immunosuppressive function, and adaptability to the TME [27]. In this study, *FCGR2B* is predominantly expressed in HGSOC‐infiltrating macrophages, and its expression is associated with patient prognosis, indicating that *FCGR2B* marks a transcriptionally distinct TAM subpopulation with potential functional relevance within the TME.

*FCGR2B* ^+^ TAMs exhibited altered intercellular communication, with enhanced interactions with T cells and transcriptional enrichment of ferroptosis‐related pathways. Hub gene network analysis highlighted central nodes, including IL1B, CXCL8, and MMP9, suggesting a potential association between inflammatory signaling and ferroptosis‐related metabolic processes in these TAMs. This result indicates that metabolic plasticity, characterized by shifted lipid and iron handling, may confer specific ferroptotic sensitivity to *FCGR2B* ^+^ TAMs, subsequently impacting their functional diversity and contribution to immune regulation in the TME landscape. IL1B, CXCL8, and MMP9 are well‐established mediators of inflammatory responses, and MMP9 has been linked to ferroptotic processes in various contexts [28–30]. These observations are consistent with previous reports showing that macrophage‐derived inflammatory mediators can modulate oxidative stress and iron metabolism within the TME, thereby potentially influencing tumor progression and immune regulation [31, 32]. Furthermore, ferroptosis cues within the TME have been shown to influence macrophage polarization, immune activation, and the clearance of dying cells in a context‐dependent manner [33–35], highlighting the dynamic interplay between ferroptosis and TAM function. Emerging evidence indicates a complex bidirectional interplay between ferroptosis and tumor‐infiltrating immune cells. IFN‐*γ* secreted by immune cells triggers the JAK‐STAT pathway in tumor cells to suppress system XC^−^ (SLC7A11/SLC3A2) expression, thereby impairing cystine uptake and inducing intercellular ferroptosis via exacerbated lipid peroxidation [36–38]. Conversely, ferroptosis can reciprocally impair immune function, as localized ferroptotic events elevate oxidative stress and the lipid metabolic burden within the TME. This predisposes CD8^+^ T cells to ferroptotic exhaustion, thereby compromising their antitumor efficacy [36]. This reciprocal regulation suggests that ferroptosis functions not merely as a mode of cell death but as a dynamic bridge between immune functionality and tumor responsiveness, implying that ferroptosis‐related mechanisms may significantly impact the efficacy of immunotherapeutic strategies.

Iron availability is a key regulator of TAM function [39]. Altered iron metabolism is a hallmark of cancer, and macrophages contribute to iron homeostasis within the TME [40, 41]. Consistent with previous observations in multiple cancer contexts, M2‐like TAMs display reduced ferrous iron levels compared with M1‐like counterparts, and tumor cells can compete for iron through transferrin receptor (TFRC) overexpression, thereby driving immunosuppressive M2 polarization [42]. These data support the concept that iron metabolism and ferroptosis‐related pathways are intimately linked to TAM heterogeneity and functional plasticity, providing a mechanistic basis for the transcriptional enrichment observed in *FCGR2B* ^+^ TAMs in HGSOC.

Beyond its utility as a phenotypic marker, *FCGR2B* is the constitutive inhibitory Fc*γ* receptor. Mechanistically, its intracytoplasmic ITIM recruits inhibitory phosphatases (*e.g.*, SHIP1/2) upon ligation to antagonize ITAM‐mediated signaling, thereby constraining inflammatory cascades and maintaining the immunosuppressive state of TAMs [43]. Immunometabolic research further demonstrates that Fc*γ* receptor signaling drives the reprogramming of macrophage immunometabolic circuits, such as the activation of energy metabolism pathways including glycolysis [44]. Iron metabolism and the intracellular redox environment serve as the core regulatory nodes for ferroptosis susceptibility. As immune cells with highly active iron turnover, macrophages govern cellular iron homeostasis through the integrated processes of iron uptake, sequestration, and efflux [45], which underscores that the intrinsic iron metabolic signature of macrophages likely dictates their ferroptotic response. Consequently, although direct evidence linking *FCGR2B* to iron transporters is limited, the established nexus between Fc*γ*R signaling and immunometabolic reprogramming suggests that *FCGR2B* indirectly modulates ferroptosis sensitivity. This is likely achieved through the recalibration of cellular metabolism and redox homeostasis. Stage‐specific expression of prognostic genes along macrophage differentiation trajectories, coordinated by transcription factors such as CEBPB and SPI1, further suggests that CNV and transcriptional regulation contribute to TAM functional diversity and may be associated with ferroptosis‐related metabolic programming. In HGSOC, the frequent genomic amplifications of *CRYAB*, *PLAUR*, and *EREG* likely drive their overexpression through a gene dosage effect. These upregulated factors may actively orchestrate the functional polarization of immunosuppressive TAMs, reinforcing their protumorigenic phenotype and modulating ferroptosis susceptibility. *CRYAB* serves as a pivotal regulator of cellular proteostasis and ferroptosis sensitivity under various stress conditions [46]. *PLAUR* may promote immunosuppression within the TME by orchestrating immune responses and enhancing cell migration [47]. The *EREG* signaling axis serves as a critical suppressor of ferroptosis, thereby modulating the cellular threshold for lipid peroxidation [48]. Such genomic amplification potentially intersects with transcriptional and metabolic reprogramming, providing a mechanistic foundation for the cellular heterogeneity and ferroptosis evasion observed in *FCGR2B* ^+^ TAMs within the HGSOC TME. C/EBP*β* has been experimentally shown to govern M2‐like macrophage gene expression and contribute to macrophage polarization in tumor contexts [49–51], and broader transcriptional networks involving SPI1 and other TFs orchestrate macrophage polarization in diverse tumor contexts [52–54]. Collectively, these findings integrate transcriptional, metabolic, and ferroptotic cues to provide a comprehensive view of TAM heterogeneity and function within the TME of HGSOC.

Collectively, this study supports a model in which *FCGR2B* ^+^ TAMs in HGSOC contribute to shaping a transcriptional and metabolic landscape that integrates immune regulation and iron metabolism, providing a potential mechanistic link to ferroptosis susceptibility. This highlights the importance of considering TAM heterogeneity when designing ferroptosis‐targeted therapeutic strategies, and our RSF‐based prognostic model may serve as a useful exploratory tool for patient stratification and hypothesis generation in personalized therapy development.

## 5. Limitations and Future Directions

Several limitations of this study should be acknowledged. First, the prognostic model was developed and validated primarily using public datasets and a single‐center cohort; therefore, further validation in larger, multicenter cohorts is required to assess its robustness and generalizability. Second, as this study was mainly based on bioinformatic analyses, the biological functions of the core genes identified herein remain to be validated through in vitro and in vivo experiments. Third, the absence of spatial transcriptomic data represents an important limitation, as it precludes direct validation of the spatial organization and cell–cell interactions within the TME. Future studies integrating spatially resolved transcriptomic approaches may help to elucidate the tissue architecture. In addition, although our risk score showed independent prognostic value, integrating this molecular signature with routine clinicopathological variables, such as FIGO stage and residual disease status, could further enhance its clinical applicability. Future validation in larger, prospective, multicenter cohorts is essential to confirm its stability and utility for individualized decision‐making. Moreover, our computational drug sensitivity predictions presented here are in silico findings derived from genomic profiles. These results warrant further validation in in vitro cocultures or patient‐derived organoids to assess their functional relevance and translational potential.

## 6. Conclusion

This study systematically characterized *FCGR2B*‐associated prognostic genes in HGSOC using single‐cell transcriptomics, pseudotime trajectory, regulatory network, and CNV analyses. *CRYAB*, *PLAUR*, *EREG*, and *C5AR1* showed stage‐specific expression in macrophages, coordinated by key transcription factors, and gene amplifications contributed to elevated expression. Molecular docking and drug‐gene analyses highlighted potential therapeutic compounds targeting these genes. These findings underscore their prognostic and therapeutic relevance in HGSOC and provide a basis for precision risk stratification and individualized clinical practice.

## Author Contributions

Jialu Zhou, Tao Zeng, and Yi Liu contributed equally to this work. Jialu Zhou, Tao Zeng, and Yi Liu: conceptualization, data curation, formal analysis, methodology, validation, visualization, and writing—original draft. Mingxia Ye and Mingxia Li: formal analysis, methodology, and validation. Mingxia Ye and Zhe Zhang: funding acquisition. Zhe Zhang and Yuanguang Meng: supervision, and writing—review and editing.

## Funding

No funding was received for this manuscript.

## Disclosure

All authors have read and approved the final version of the manuscript.

## Ethics Statement

The authors have nothing to report.

## Consent

The authors have nothing to report.

## Conflicts of Interest

The authors declare no conflicts of interest.

## Supporting Information

Additional supporting information can be found online in the Supporting Information section.

## Supporting information


**Supporting Information 1** Figure S1: Quality control, batch correction, and cell composition of scRNA‐seq data from HGSOC and normal ovarian tissues. (a, b) Quality control analysis of scRNA‐seq data (a) before and (b) after filtering. Violin plots display the distributions of total RNA counts per cell (nCount_RNA), the number of detected genes per cell (nFeature_RNA), and the percentage of mitochondrial genes (percent.mt). (c) JackStraw analysis for the selection of significant principal components used for downstream clustering. (d) UMAP visualization of cells before (left) and after (right) Harmony batch correction, demonstrating effective removal of batch effects. (e) Relative proportions of eight major cell types in HGSOC and normal ovarian tissues. (f) Comparison of cell type proportions between HGSOC and normal tissues. Statistical significance was assessed using the chi‐square test ( ^∗^ *p* < 0.05,  ^∗∗^ *p* < 0.01,  ^∗∗∗^ *p* < 0.001).

**Supporting Information 2** Figure S2: Key ligand–receptor interactions of macrophages in normal ovarian tissues. Bubble plot showing major ligand–receptor pairs where macrophages act as signaling sources (left) or targets (right). The color of the bubbles represents the interaction strength, and the size indicates the statistical significance (*p* value) of the interactions. Significant interactions were defined as *p* < 0.05.

**Supporting Information 3** Figure S3: HdWGCNA analysis of macrophage‐specific gene coexpression modules in GSE184880. (a) Soft‐thresholding power selection for network construction. Four panels show scale‐free topology fit index (signed *R* ^2^), mean connectivity, median connectivity, and maximum connectivity across soft‐thresholding powers (1–20). The dashed line indicates the selected optimal power (*β* = 6). (b) Hierarchical clustering tree of genes. Each branch represents a gene, with modules identified by a dynamic tree cut shown in distinct colors. The gray module contains genes that are not assigned to any other module. (c) Top 10 hub genes (highest kME) in each module. Bar plots indicate kME values; genes are ordered from lowest to highest kME. (d) UMAP visualization of module scores calculated from the Top 25 hub genes in each module. Each point represents a single cell, colored by aggregated module gene scores, showing the distribution of highly connected genes across macrophage subpopulations. (e) Module–module correlation heatmap. Correlation coefficients between modules are indicated by colored circles (purple: positive correlation; green: negative correlation). The circle shape and orientation represent the correlation direction, with compression toward the diagonal indicating a positive or negative correlation, and a perfect circle indicating no correlation. Circle size reflects correlation magnitude, and × marks indicate missing or undefined correlations.

**Supporting Information 4** Figure S4: Schoenfeld residual analysis for Cox proportional hazards assumption. (a) Scatter plots showing Schoenfeld residuals versus survival time for *EREG*, *C5AR1*, *CRYAB*, and *PLAUR*, with trend lines indicating no significant time‐dependent deviation (*p* > 0.05), confirming the proportional hazards assumption. (b) Multivariable Cox regression analysis demonstrating that the RSF‐derived risk score remains an independent prognostic factor after adjustment for age and FIGO stage (HR = 1.026, 95% CI: 1.021–1.030, *p* < 0.001). Statistical significance is indicated as follows:  ^∗^ *p* < 0.05,  ^∗∗^ *p* < 0.01,  ^∗∗∗^ *p* < 0.001; N.S., nonsignificant.

**Supporting Information 5** Figure S5: Single‐cell pseudotime analysis of macrophage differentiation in HGSOC. (a) UMAP visualization of macrophage subclusters in the GSE184880 dataset. Each point represents a single cell, colored by unsupervised clustering. (b, c) Pseudotime trajectory of macrophages inferred using Monocle. Colors indicate (b) the sample group in the left panel and (c) the macrophage cluster in the right panel.

**Supporting Information 6** Figure S6: Genomic and CNV analysis of prognostic genes. (a) The scatter plots illustrate the correlation between copy number variation and gene expression levels for four prognostic genes. (b) Somatic mutation profiles in the high‐risk group, showing the most frequently mutated genes, including TP53, TTN, and FLG2. (c) Somatic mutation profiles in the low‐risk group, with TP53, TTN, and CSMD3 as the most frequently mutated genes. (d) Missense mutations detected in *C5AR1* among the four prognostic genes. (e) TMB comparison between high‐risk and low‐risk groups, showing no significant differences (*p* > 0.05). Statistical significance is indicated as follows:  ^∗^ *p* < 0.05,  ^∗∗^ *p* < 0.01,  ^∗∗∗^ *p* < 0.001; N.S., nonsignificant.

**Supporting Information 7** Table S1: Cell type annotation of single‐cell RNA‐seq in HGSOC.

**Supporting Information 8** Table S2: Differentially expressed genes between HGSOC and normal samples identified from bulk RNA‐seq data.

**Supporting Information 9** Table S3: Differentially expressed genes between *FCGR2B*‐high and *FCGR2B*‐low groups identified from bulk RNA‐seq data.

**Supporting Information 10** Table S4: Differentially expressed genes between HGSOC and normal macrophages identified from single‐cell RNA‐seq data.

**Supporting Information 11** Table S5: Macrophage‐related gene modules identified by hdWGCNA analysis.

**Supporting Information 12** Table S6: Ferroptosis‐related genes curated from GeneCard.

**Supporting Information 13** Table S7: Univariate Cox proportional hazards regression analysis of 26 candidate genes for overall survival.

**Supporting Information 14** Table S8: Baseline clinical characteristics of high‐risk and low‐risk groups.

**Supporting Information 15** Table S9: Gene set enrichment analysis (GSEA) for the four prognostic genes.

**Supporting Information 16** Table S10: Molecular docking analysis between four prognostic genes and candidate compounds.

**Supporting Information 17** Table S11: Comparison of estimated half‐maximal inhibitory concentration (IC50) for common chemotherapeutic drugs between high‐ and low‐risk groups.

## Data Availability

All public datasets utilized in this study are publicly accessible. The TCGA‐OV transcriptomic and clinical data were retrieved from the GDC Data Portal (https://portal.gdc.cancer.gov/). Data from the GTEx project were obtained via the GTEx Portal (https://www.gtexportal.org/home/). The bulk and single‐cell RNA sequencing datasets, specifically GSE102073 and GSE184880, are available through the NCBI Gene Expression Omnibus (GEO) (https://www.ncbi.nlm.nih.gov/geo/). The comprehensive list of FRGs was compiled by searching the GeneCards database (https://www.genecards.org/). The data that support the findings of this study are available from the corresponding authors upon reasonable request.
