# Supplementary figures and images for: FCGR2B + Macrophages as a Critical Node Linking Ferroptosis and Immunosuppression: A Multiomics Framework for Prognosis and Therapy in High‐Grade Serous Ovarian Cancer
Source: Hum Mutat. 2026 Apr 6;2026:8027584. doi: 10.1155/humu/8027584 (PMC13054137; doi:10.1155/humu/8027584)

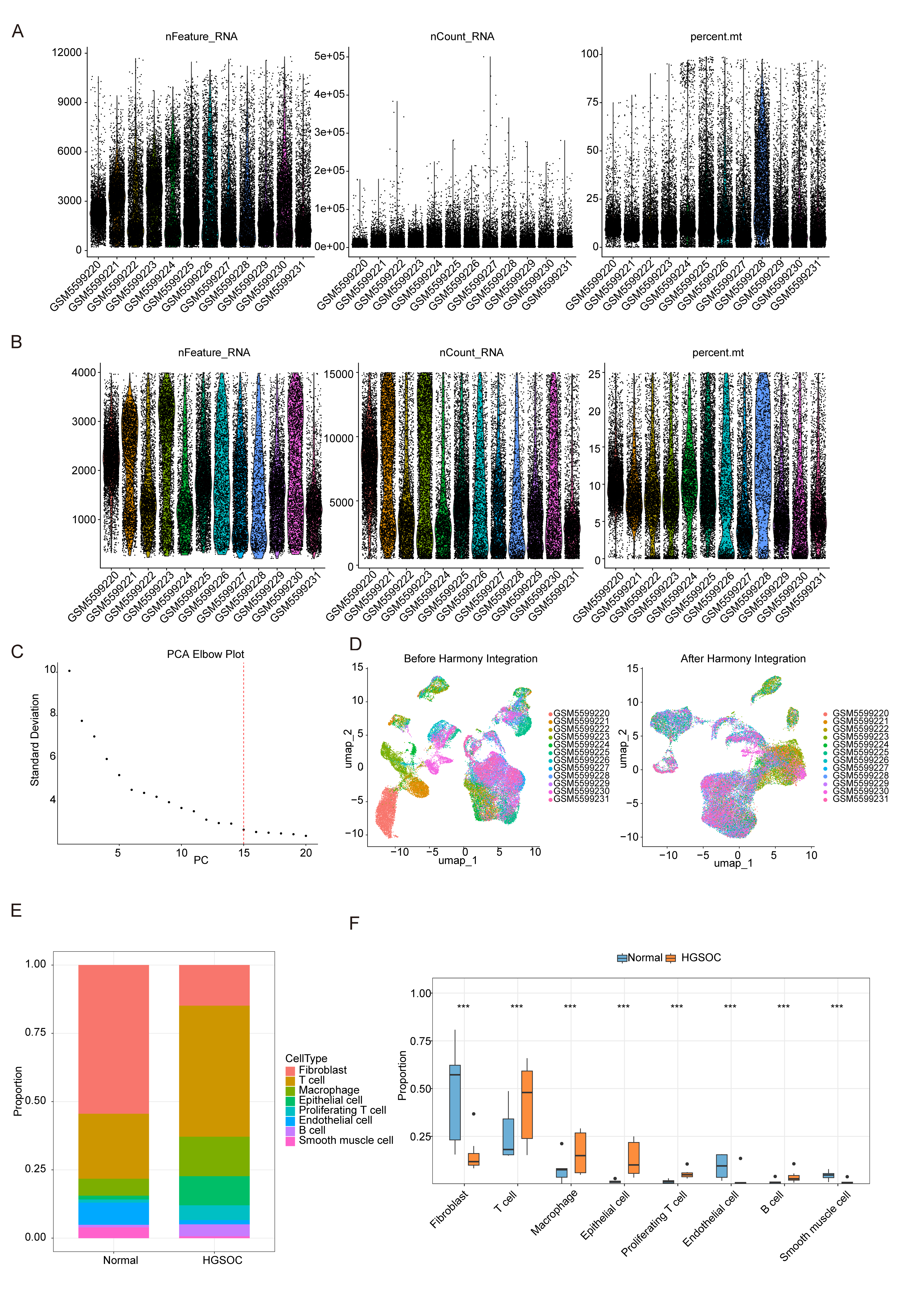

Supplement: Supplementary file 1 — Supporting Information 1 Figure S1: Quality control, batch correction, and cell composition of scRNA‐seq data from HGSOC and normal ovarian tissues. (a, b) Quality control analysis of scRNA‐seq data (a) before and (b) after filtering. Violin plots display the distributions of total RNA counts per cell (nCount_RNA), the number of detected genes per cell (nFeature_RNA), and the percentage of mitochondrial genes (percent.mt). (c) JackStraw analysis for the selection of significant principal components used for downstream clustering. (d) UMAP visualization of cells before (left) and after (right) Harmony batch correction, demonstrating effective removal of batch effects. (e) Relative proportions of eight major cell types in HGSOC and normal ovarian tissues. (f) Comparison of cell type proportions between HGSOC and normal tissues. Statistical significance was assessed using the chi‐square test ( ∗ p < 0.05, ∗∗ p < 0.01, ∗∗∗ p < 0.001). [file HUMU-2026-8027584-s001.tif]

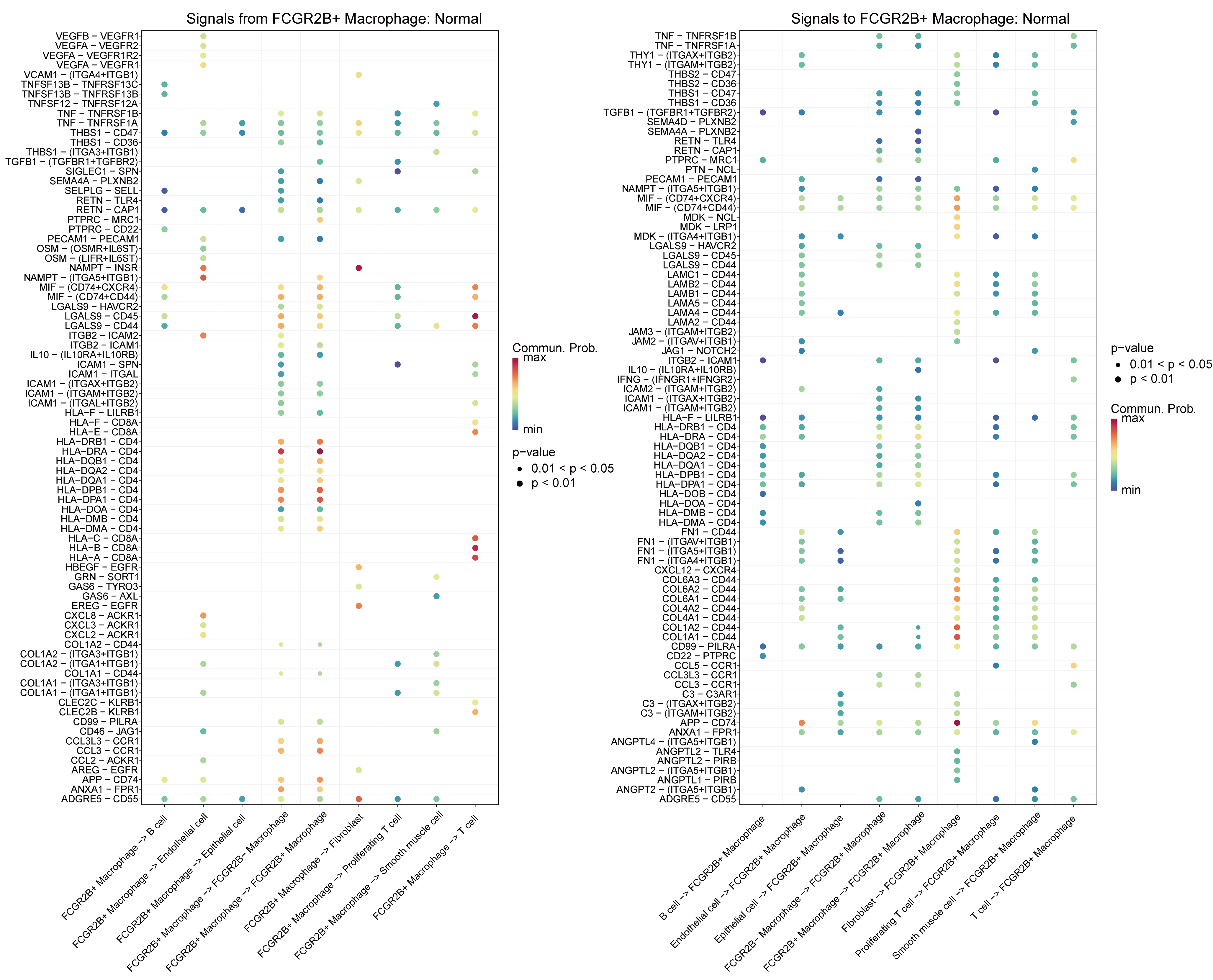

Supplement: Supplementary file 2 — Supporting Information 2 Figure S2: Key ligand–receptor interactions of macrophages in normal ovarian tissues. Bubble plot showing major ligand–receptor pairs where macrophages act as signaling sources (left) or targets (right). The color of the bubbles represents the interaction strength, and the size indicates the statistical significance (p value) of the interactions. Significant interactions were defined as p < 0.05. [file HUMU-2026-8027584-s017.tif]

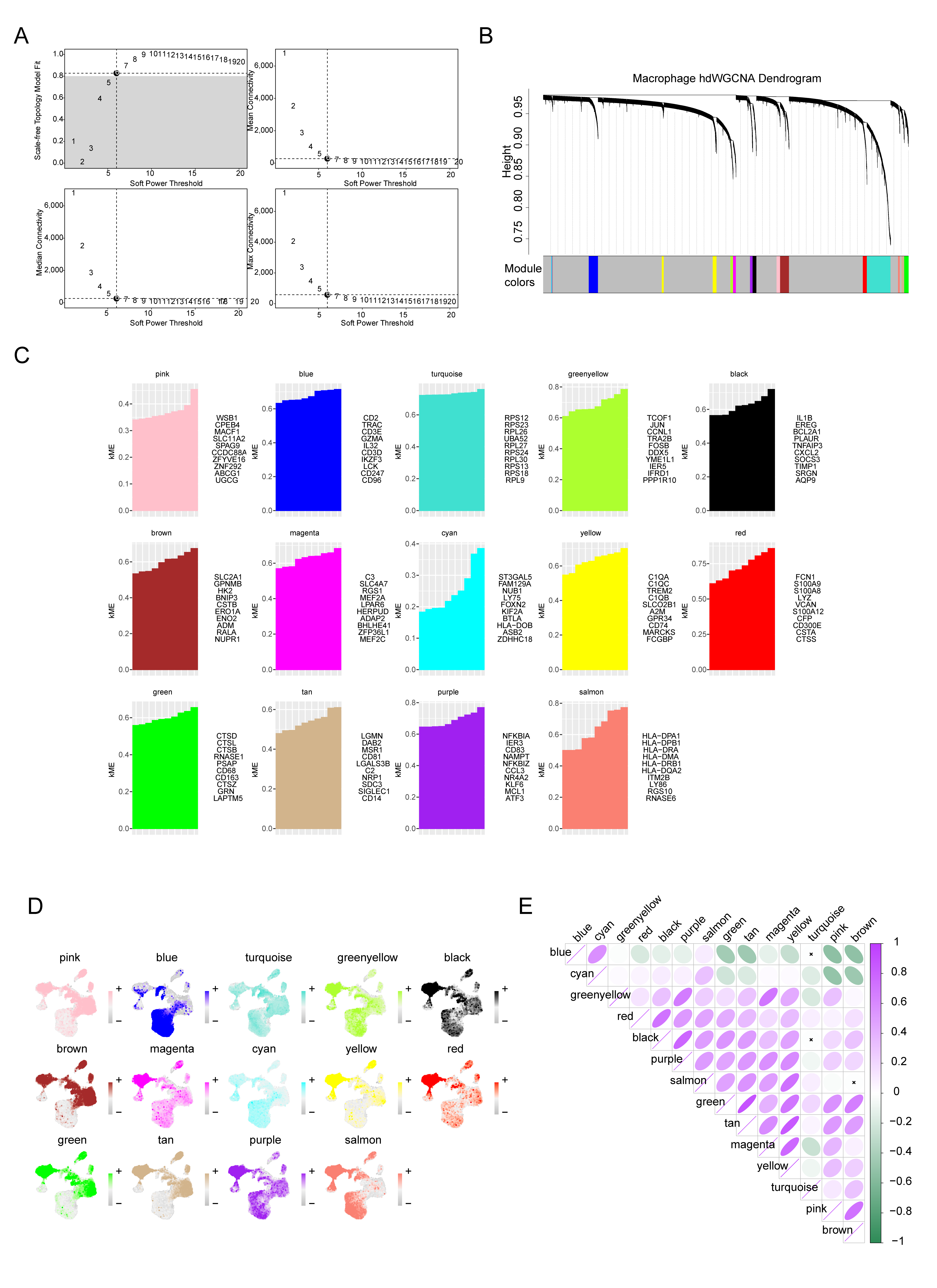

Supplement: Supplementary file 3 — Supporting Information 3 Figure S3: HdWGCNA analysis of macrophage‐specific gene coexpression modules in GSE184880. (a) Soft‐thresholding power selection for network construction. Four panels show scale‐free topology fit index (signed R 2), mean connectivity, median connectivity, and maximum connectivity across soft‐thresholding powers (1–20). The dashed line indicates the selected optimal power (β = 6). (b) Hierarchical clustering tree of genes. Each branch represents a gene, with modules identified by a dynamic tree cut shown in distinct colors. The gray module contains genes that are not assigned to any other module. (c) Top 10 hub genes (highest kME) in each module. Bar plots indicate kME values; genes are ordered from lowest to highest kME. (d) UMAP visualization of module scores calculated from the Top 25 hub genes in each module. Each point represents a single cell, colored by aggregated module gene scores, showing the distribution of highly connected genes across macrophage subpopulations. (e) Module–module correlation heatmap. Correlation coefficients between modules are indicated by colored circles (purple: positive correlation; green: negative correlation). The circle shape and orientation represent the correlation direction, with compression toward the diagonal indicating a positive or negative correlation, and a perfect circle indicating no correlation. Circle size reflects correlation magnitude, and × marks indicate missing or undefined correlations. [file HUMU-2026-8027584-s003.tif]

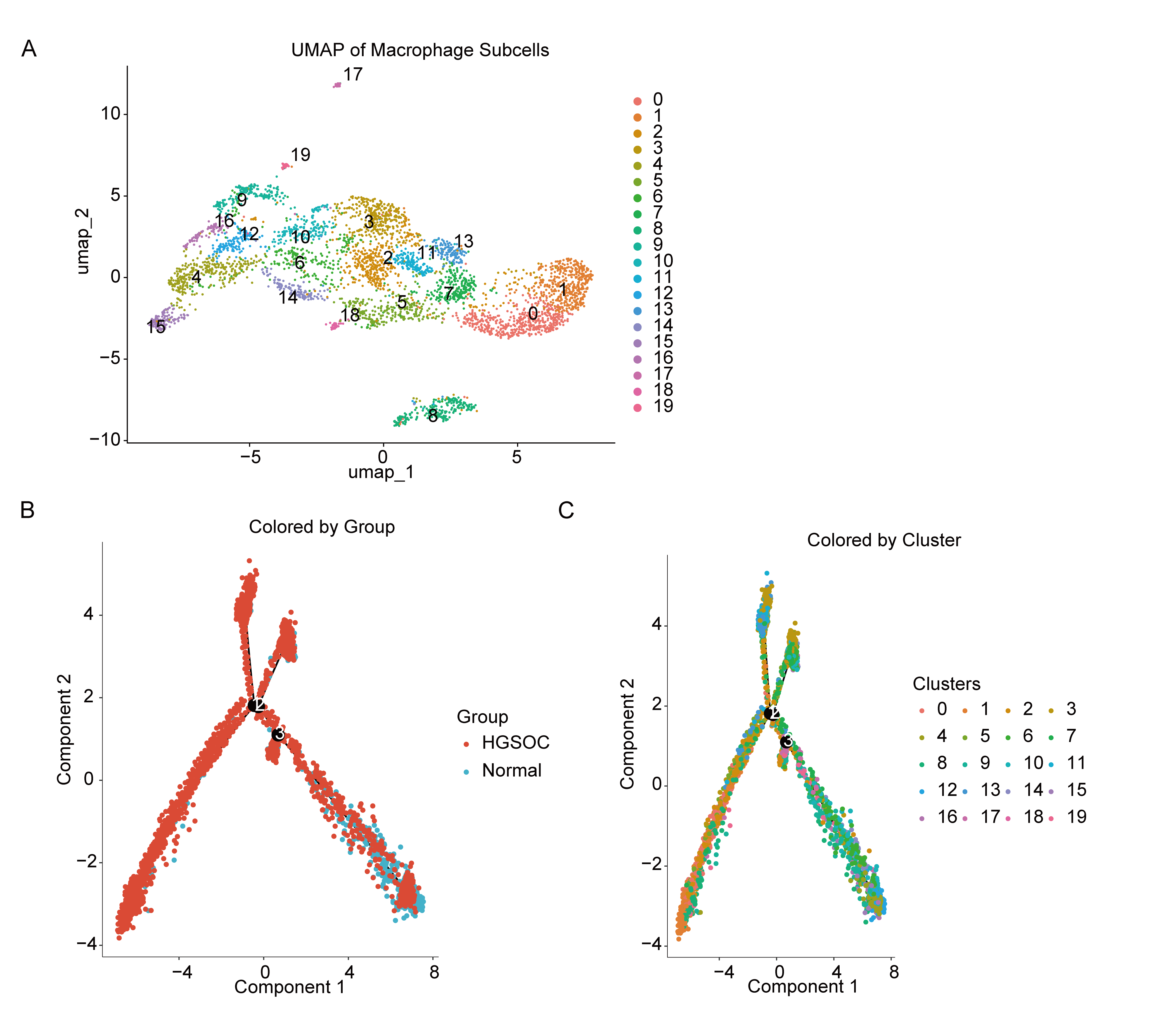

Supplement: Supplementary file 5 — Supporting Information 5 Figure S5: Single‐cell pseudotime analysis of macrophage differentiation in HGSOC. (a) UMAP visualization of macrophage subclusters in the GSE184880 dataset. Each point represents a single cell, colored by unsupervised clustering. (b, c) Pseudotime trajectory of macrophages inferred using Monocle. Colors indicate (b) the sample group in the left panel and (c) the macrophage cluster in the right panel. [file HUMU-2026-8027584-s005.tif]
